# Nrf2 and STAT3 Pathways in Protecting Against Methylmercury-Induced Oxidative Stress in Hypothalamic Neuronal GT1-7 Cells

**DOI:** 10.1007/s11064-026-04898-1

**Published:** 2026-10-08

**Authors:** Beatriz Ferrer, Alexey A. Tinkov, Abel Santamaria, João Batista Rocha, Anatoly V. Skalny, Aaron B. Bowman, Michael Aschner

**Affiliations:** 1 Department of Molecular Pharmacology, Albert Einstein College of Medicine, 1300 Morris Park Avenue, Bronx, NY 10461 USA; 2 Institute of Bioelementology, Orenburg State University, Orenburg, Russia; 3 Laboratory of Molecular Dietetics, IM Sechenov First Moscow State Medical University, Moscow, Russia; 4 Laboratory of Ecobiomonitoring and Quality Control, Yaroslavl State University, Yaroslavl, Russia; 5 Laboratorio de Nanotecnología y Nanomedicina, Departamento de Atención a la Salud, Universidad Autónoma Metropolitana-Xochimilco, Mexico City, 04960 Mexico; 6 Facultad de Ciencias, Universidad Nacional Autónoma de México, Mexico City, 04510 Mexico; 7 Departamento de Bioquímica e Biologia Molecular, Universidade Federal de Santa Maria, Santa Maria, Rio Grande do Sul Brazil; 8 Department of Medical Elementology, Peoples’ Friendship University of Russia (RUDN University), Moscow, 117198 Russia; 9 School of Health Sciences, Purdue University, West Lafayette, IN 47907 USA

**Keywords:** Methylmercury, Heavy metals, Oxidative stress, Inflammation

## Abstract

Oxidative stress plays a critical role in the methylmercury (MeHg) neurotoxicity, as MeHg induces reactive oxygen species (ROS) generation and impairs antioxidant defense systems. However, the precise molecular mechanisms underlying MeHg-induced neurotoxicity remain elusive. The nuclear factor erythroid 2-related factor 2 (Nrf2) activates antioxidant defense genes in response to acute MeHg exposure. Similarly, the signal transducer and activator of transcription 3 (STAT3), a transcription factor involved in cell growth and anti-apoptotic genes, also contributes to maintaining redox homeostasis. Here, we sought to investigate the roles of Nrf2 and STAT3 in mouse hypothalamic neuronal GT1-7 cells exposed to MeHg. We inhibited STAT3 either pharmacologically with AG490 (a JAK2 inhibitor) or C188-9 (a small-molecule STAT3 SH2 domain inhibitor) or genetically using a lentivirus carrying STAT3 shRNA (vs. scramble control), in GT1-7 cells exposed to 5 µM MeHg. To further dissect the role of Nrf2, the pathway was pharmacologically inhibited using brusatol or activated with sulforaphane (SFN) in GT1-7 cells co-exposed to 5 µM MeHg. Changes in cell viability, reactive species production, and protein and gene expression were analyzed. Our findings reveal that deficits of either the Nrf2 or STAT3 pathways exacerbated MeHg-induced toxicity. Moreover, in MeHg-exposed cells, Nrf2 ablation led to increased STAT3 activity, suggesting a reciprocal and potentially compensatory responses between these two pathways in protecting against MeHg-induced oxidative stress. Our novel findings provide crucial understanding of the molecular mechanisms underlying MeHg neurotoxicity and offer insights into potential therapeutic strategies for mitigating acute MeHg-induced neurotoxicity.

**Graphical Abstract:**

**Figure:**
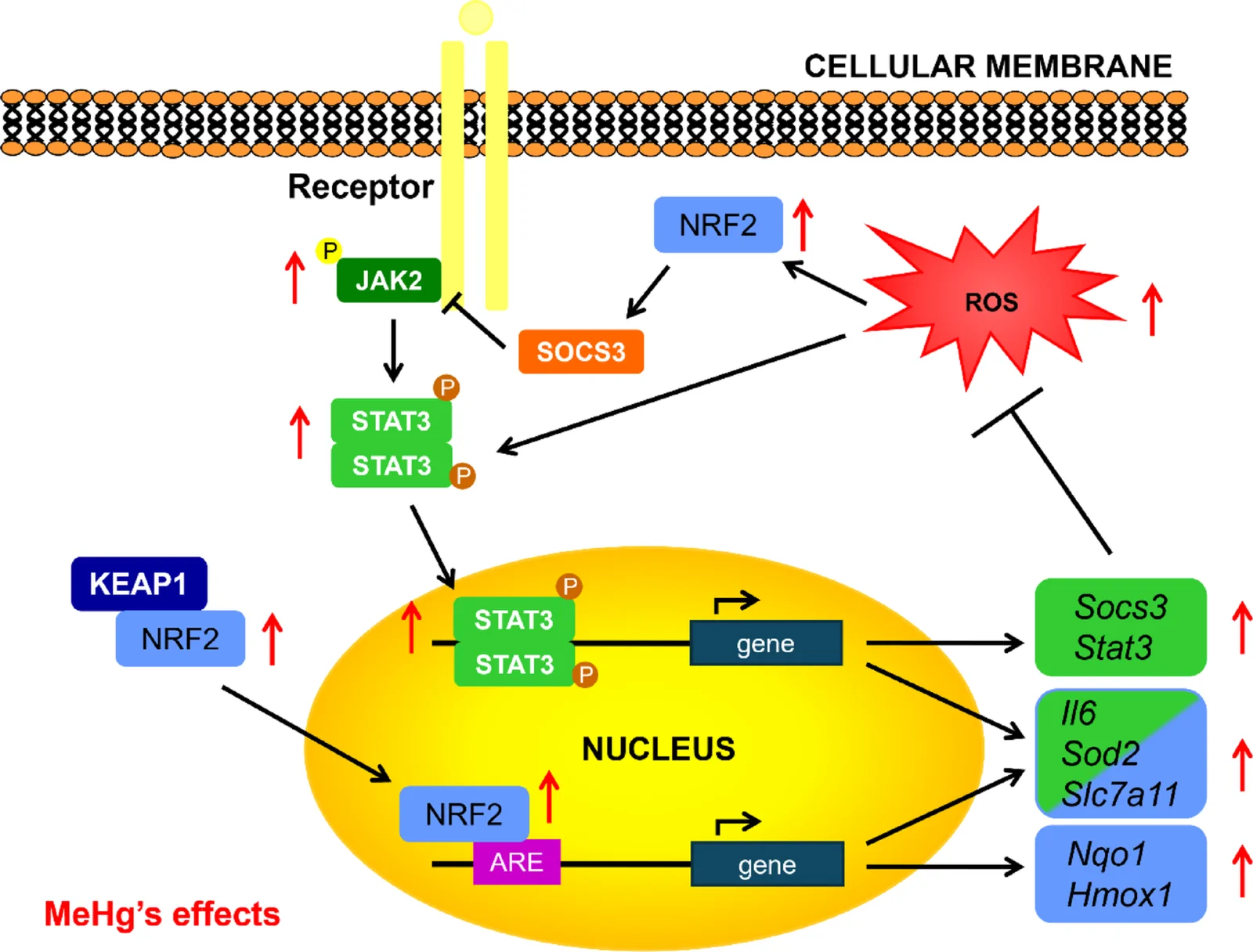

**Supplementary Information:**

The online version contains supplementary material available at https://doi.org/10.1007/s11064-026-04898-1.

## Introduction

Oxidative stress arises from an imbalance in the cellular redox state. Under physiological conditions, the generation of reactive oxygen species (ROS) is tightly regulated by antioxidant defense systems. However, when ROS levels overwhelm the antioxidant defenses due to impairment in antioxidant enzymes’ activity or excessive ROS production, oxidative stress ensues [1]. ROS are highly reactive molecules that can damage proteins, nucleic acids, and lipids, ultimately leading to cellular death [2].

Nuclear factor erythroid 2-related factor 2 (Nrf2) is a master regulator of cellular responses to oxidative stress [3, 4]. Nrf2 is a transcription factor encoded by the *Nfe2l2* gene and belongs to the cap ‘n’ collar family, characterized by a basic leucine zipper DNA-binding domain in its structure. Under basal conditions, Nrf2 resides in the cytoplasm, where it is bound to Kelch-like ECH-associated protein 1 (KEAP1). KEAP1 acts as an adaptor protein for the Cul3-Rbx1 E3 ubiquitin ligase complex, promoting Nrf2 ubiquitination and subsequent proteasomal degradation. KEAP1 contains several cysteine residues that can be modified by electrophilic compounds and ROS. Under oxidative stress conditions, these modifications disrupt the Nrf2-KEAP1 interaction, leading to Nrf2 nuclear translocation [5]. Once in the nucleus, Nrf2 binds to the antioxidant responsive element (ARE) and activates the expression of antioxidant and phase-II detoxifying enzymes, such as NAD(P)H dehydrogenase quinone 1 (NQO1), heme oxygenase 1 (HO-1), and solute carrier family 7 member 11 (SLC7A11) [6–8]. Both in vitro and in vivo experimental models have demonstrated that genetic or pharmacological activation of Nrf2 provides protection against oxidative stress [9, 10], whereas Nrf2 depletion exacerbates oxidative stress-induced cell damage [11, 12].

Signal transducer and activator of transcription 3 (STAT3) has emerged as a key regulator of cellular redox balance, influencing both ROS production and oxidative metabolism. Constitutively active STAT3 protects cells from apoptosis induced by ROS production [13]. Additionally, genetic or pharmacological depletion of STAT3 is associated with mitochondrial dysfunction and elevated ROS levels [14–16]. Furthermore, STAT3 can also provide antioxidant protection by upregulating specific antioxidant genes. For instance, STAT3 binds to the promoter of superoxide dismutase 2 (SOD2) and induces its gene transcription in the mouse brain and primary cortical neurons, potentially reducing ROS generation [17]. Additionally, STAT3 enhances the expression of *Slc7a11* by recruitment to a gamma-activated site (GAS) motif present in *Slc7a11* promoter, reducing intracellular ROS levels in human breast cancer cells [18].

In addition to its role in redox homeostasis, STAT3 itself is susceptible to the effects of oxidative stress. However, the precise impact of ROS on STAT3 activation remains controversial. While some studies have reported that oxidative stress impairs STAT3 transcriptional activity [19–21], others have observed increased STAT3 phosphorylation and nuclear translocation following exposure to various oxidative stimuli [22–24]. Furthermore, the activation of STAT3 by ROS is inhibited in cells treated with ROS scavengers, such as N-acetyl L cysteine [25], further supporting a role for ROS in inducing STAT3 activity. However, the reasons for these discrepancies remain unclear and require further investigation.

Environmental toxicants such as pesticides, air pollutants, and heavy metals can disrupt mitochondrial function and generate ROS. Oxidative stress is a primary neurotoxic mechanism of methylmercury (MeHg). MeHg is a naturally occurring environmental pollutant, generated from sources such as volcanoes and forest fires. However, increased global industrialization and technological advancements, such as mining, coal burning, and waste incinerators, have led to greater mercury release into the environment. Once mercury enters aquatic systems, it is biomethylated by sulfate-reducing microorganisms to form MeHg [26]. MeHg then bioaccumulates within the food chain, reaching high concentrations in predatory fish species. Consequently, the primary source of human MeHg exposure is the consumption of contaminated fish and shellfish. Upon ingestion, MeHg is widely distributed throughout the body, reaching the brain, which is particularly vulnerable, especially during developmental stages [27]. Several epidemiological studies have demonstrated that prenatal exposure to MeHg is associated with impaired neurobehavioral development in children. Specifically, children exposed to maternal MeHg during gestation have been reported to exhibit locomotor, cognitive, and to a lesser extent, visual deficits [28–33]. Although the neurotoxicity of MeHg has been well-documented for decades, it continues to pose a significant risk to human health.

Alterations in redox homeostasis are key consequences of MeHg-induced neurotoxicity. MeHg disrupts mitochondrial membrane potential, triggering the formation of ROS [34]. Additionally, as a soft electrophile, MeHg readily binds to thiol and selenol groups within proteins, disrupting their structure and function [35]. Several antioxidant proteins have thiol and selenol groups in their structures, and when MeHg binds to them, it impairs antioxidant defense systems. The combined increase in ROS production and compromised antioxidant defenses ultimately results in MeHg-induced oxidative stress.

MeHg has also been shown to activate Nrf2, leading to the induction of downstream antioxidant genes [10, 36]. Interestingly, Nrf2 deficiency increases vulnerability to MeHg, while induction of Nrf2 pathway confers protection against MeHg [9, 10, 37]. Similarly, in vitro and in vivo studies have demonstrated that MeHg activates JAK2/STAT3 signaling pathway [38–40].

Our group has previously demonstrated that pharmacological inhibition of STAT3 activation exacerbates MeHg-induced oxidative stress [41]. Several studies suggest an interplay between Nrf2 and STAT3 [42–46]; however, the mechanistic nature of this interaction is unclear. This study aimed to investigate the relationship between Nrf2 and STAT3, specifically in the context of MeHg exposure. Our new findings provide support for a model in which STAT3 inhibition is associated with increased Nrf2 abundance in the nuclear fraction and subsequent upregulation of its downstream target genes. Conversely, we report that pharmacological inhibition of Nrf2 led to an increase in MeHg-induced STAT3 phosphorylation and gene transcription. Taken together, our findings support a compensatory mechanisms between Nrf2 and STAT3 in counteracting MeHg-induced oxidative stress. These findings have important implications for the development of novel therapeutic strategies to prevent acute MeHg-induced neurotoxicity and may also provide insights into other neurological disorders where oxidative stress plays a critical role in their pathogenesis.

## Materials and Methods

### Cell Culture

Mouse hypothalamic neuronal GT1-7 cells (RRID: CVCL_0281; kindly provided by Dr Pamela Mellon, University of California, San Diego) [47] were cultured in Dulbecco’s Modified Eagle’s Medium (DMEM) (Gibco™. cat. no. 11995040) supplemented with 10% heat-inactivated fetal bovine serum (Gibco™, cat. no. 10438034) and 1% penicillin/streptomycin (Gibco™, cat. no. 15140148). Cells were maintained at 37 °C in a humidified atmosphere containing 5% CO_2_ and used for experiments up to a maximum of 35 passages to ensure cell line integrity and reproducibility of results. GT1-7 cell line used in this study are not listed as a commonly misidentified cell line by the International Cell Line Authentication Committee (ICLAC) database. Authentication of the GT1-7 cell line was verified based on its characteristic morphology and growth behavior prior to experimentation.

293T/17 cells (RRID: CVCL_1926; ATCC, cat.no. CRL-11268) were cultured in Dulbecco’s Modified Eagle’s Medium (DMEM) (Gibco™. cat. no. 11995040) supplemented with 10% heat-inactivated fetal bovine serum (Gibco™, cat. no. 10438034) and 1% penicillin/streptomycin (Gibco™, cat. no. 15140148). Cells were maintained at 37 °C in a humidified atmosphere containing 5% CO_2_.

No randomization or blind procedures were performed during the experiments involving GT1-7 cells.

### Generation of STAT3 Knockdown GT1-7 Cells

Lentiviral vector plasmids encoding murine STAT3 shRNA (cat. no. RMM3981-97059843, clone ID TRCN0000071456) or scramble shRNA (RRID: Addgene_1864) were packaged in 293T/17 cells (ATCC, cat.no. CRL-11268) by co-transfection with packaging plasmids psPAX2 (RRID: Addgene_12260) and pCMV-VSV-G (RRID: Addgene_8454), using PolyFect^®^Transfection Reagent (Qiagen cat.no. 301105) according to the manufacturer’s protocol. The shRNA sequence used to knockdown STAT3 was: 5’-CCTGAGTTGAATTATCAGCTT-3’.

GT1-7 cells were plated and infected with lentiviral particles by spinfection, where plates were centrifuged at 2000 rpm for 60 min at 25–32 °C in the presence of 8 µg/mL Polybrene transfection reagent (Millipore-Sigma, cat. no. TR-10003-G) to enhance transduction efficiency. After infection, the medium was replaced with complete DMEM containing 2.5 µg/ml puromycin (Gibco™, cat. no. A11138-03) to select for successfully transduced cells. The efficiency of STAT3 knockdown was validated by Western blot and qPCR.

### Methylmercury (MeHg) Exposure

**Fig. 1:**
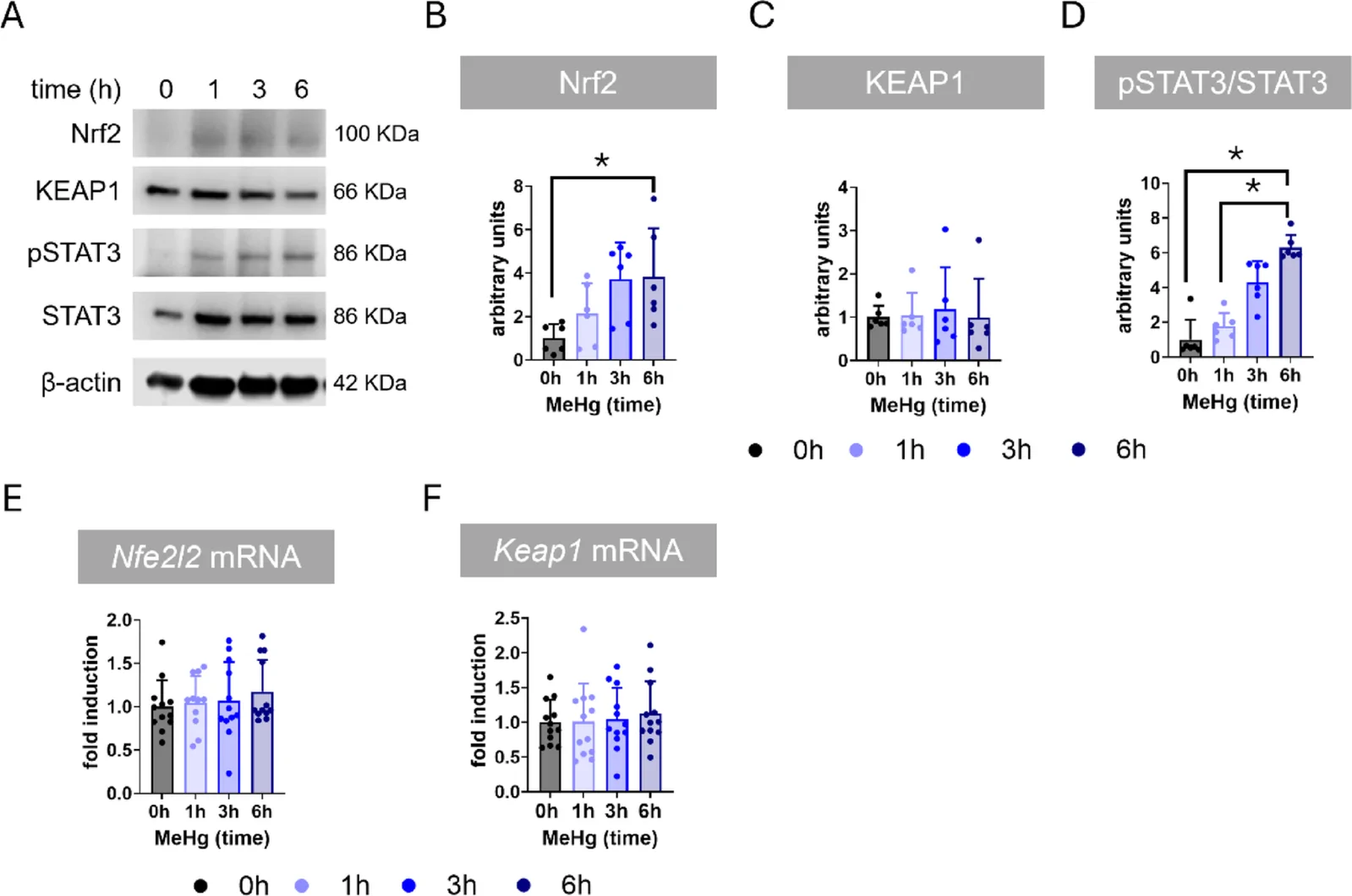
MeHg induces a time-dependent increase in Nrf2 and phospho-STAT3 protein levels in mouse hypothalamic neuronal GT1-7 cells. GT1-7 hypothalamic cells were exposed to 5 µM MeHg for 0, 1, 3, or 6 h and protein and gene expression were evaluated using western blot and qPCR, respectively. **A** Representative western blot bands of Nrf2, KEAP1, phospho-STAT3 (Y705), total STAT3, and β-actin. **B** Densitometry quantification of Nrf2 (*n* = 6 independent cell culture preparations). **C** Densitometry quantification of KEAP1 (*n* = 6 independent cell culture preparations). **D** Densitometry quantification of the phospho-STAT3/STAT3 ratio protein levels (*n* = 6 independent cell culture preparations). **E** qPCR analysis of *Nfe2l2* mRNA levels (*n* = 11–12 independent cell culture preparations). **F** qPCR analysis of *Keap1* mRNA levels (*n* = 12 independent cell culture preparations). Data are expressed as mean ± SD. * indicates *p* < 0.05

**Fig. 2:**
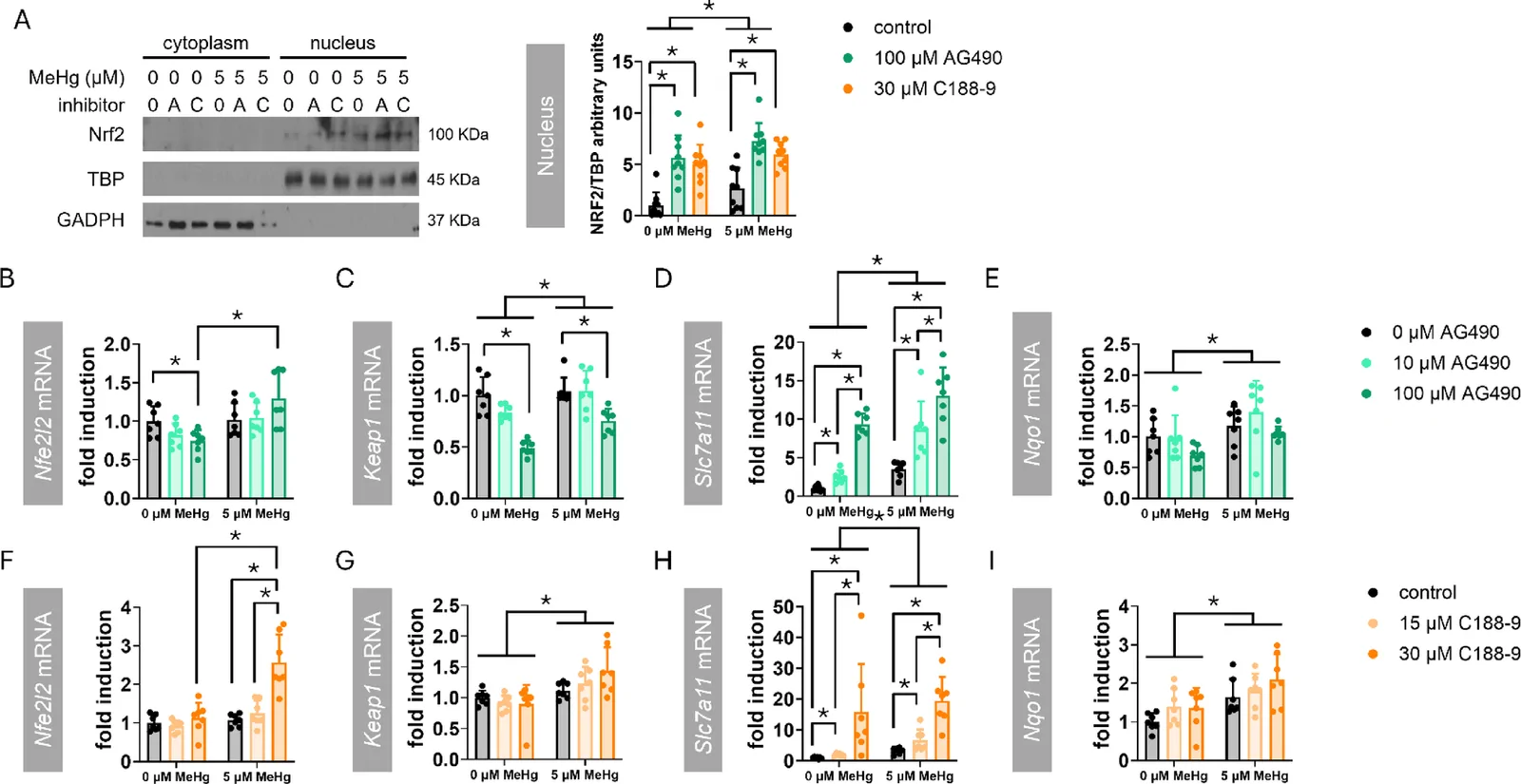
Inhibition of STAT3 enhances MeHg-induced Nrf2 activity in mouse hypothalamic neuronal GT1-7 cells. **A** Representative western blot analysis of Nrf2 protein levels in cytoplasmic and nuclear fractions, with densitometry quantification of nuclear Nrf2 protein levels in GT1-7 cells pre-treated with 100 µM AG490 or 30 µM C188-9 for 1 h, followed by co-exposure to 5 µM MeHg for 6 h (*n* = 9 independent cell culture preparations). **B** qPCR analysis of *Nfe2l2* mRNA levels in GT1-7 cells treated with 10 or 100 µM AG490 for 1 h, followed by co-exposure to 5 µM MeHg for 6 h (*n* = 7 independent cell culture preparations). **C** qPCR analysis of *Keap1* mRNA levels in GT1-7 cells treated with 10 or 100 µM AG490 for 1 h, followed by co-exposure to 5 µM MeHg for 6 h (*n* = 7 independent cell culture preparations). **D** qPCR analysis of *Slc7a11* mRNA levels in GT1-7 cells treated with 10 or 100 µM AG490 for 1 h, followed by co-exposure to 5 µM MeHg for 6 h (*n* = 7 independent cell culture preparations). **E** qPCR analysis of *Nqo1* mRNA levels in GT1-7 cells treated with 10 or 100 µM AG490 for 1 h, followed by co-exposure to 5 µM MeHg for 6 h (*n* = 7 independent cell culture preparations). **F** qPCR analysis of *Nfe2l2* mRNA levels in GT1-7 cells treated with 15 or 30 µM C188-9 for 1 h, followed by co-exposure to 5 µM MeHg for 6 h (*n* = 7 independent cell culture preparations).) qPCR analysis of *Keap1* mRNA levels in GT1-7 cells treated with 15 or 30 µM C188-9 for 1 h, followed by co-exposure to 5 µM MeHg for 6 h (*n* = 7 independent cell culture preparations). **H** qPCR analysis of *Slc7a11* mRNA levels in GT1-7 cells treated with 15 or 30 µM C188-9 for 1 h, followed by co-exposure to 5 µM MeHg for 6 h (*n* = 6–7 independent cell culture preparations). **I** qPCR analysis of *Nqo1* mRNA levels in GT1-7 cells treated with 15 or 30 µM C188-9 for 1 h, followed by co-exposure to 5 µM MeHg for 6 h (*n* = 7 independent cell culture preparations). Data are expressed as mean ± SD. * indicates *p* < 0.05

**Fig. 3:**
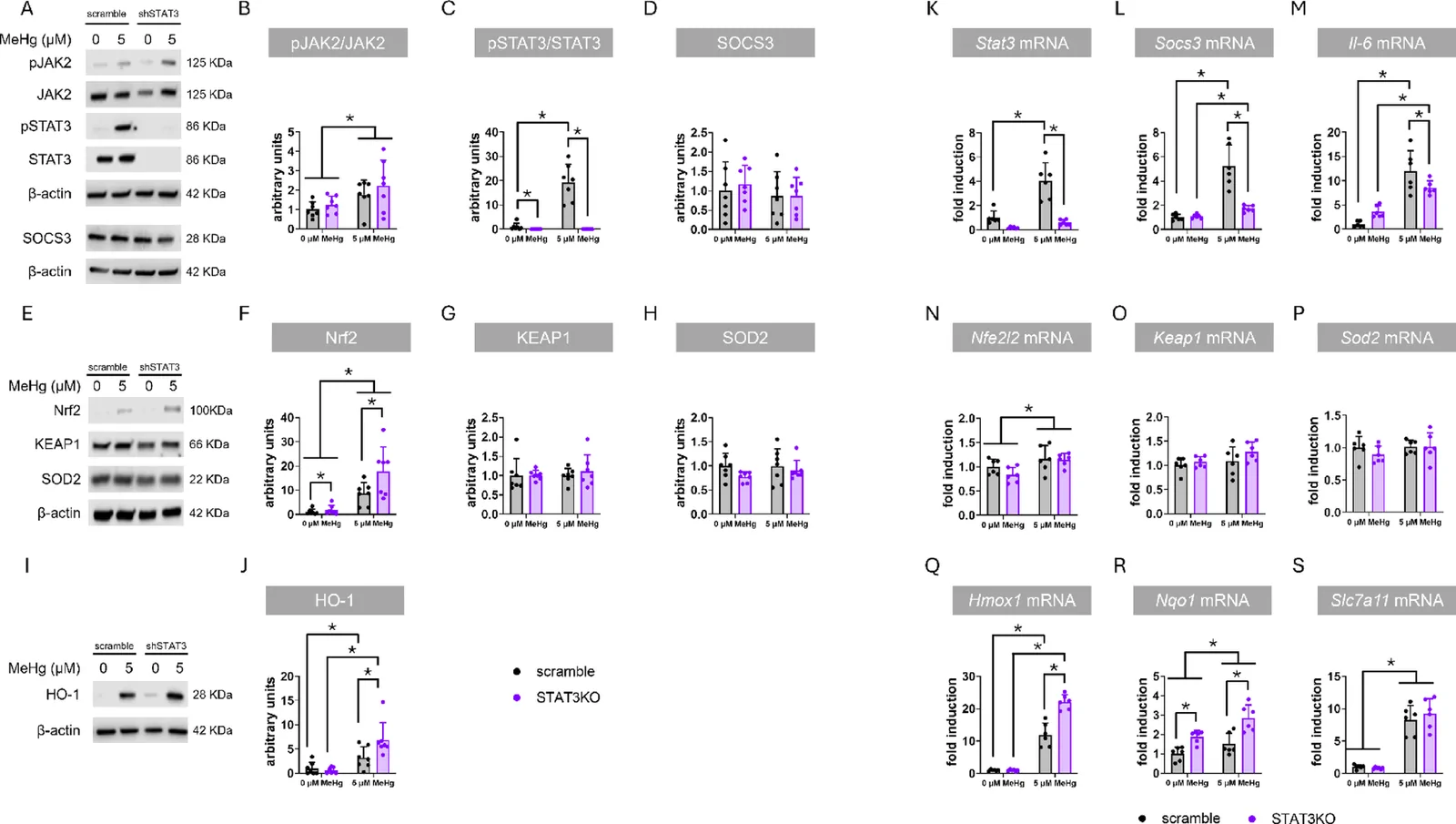
STAT3 knockdown enhances MeHg-induced Nrf2 activity in mouse hypothalamic neuronal GT1-7 cells. STAT3 expression was genetically silenced in GT1-7 cells by infection with a lentivirus encoding an shRNA targeting STAT3. GT1-7 cells infected with a lentivirus carrying a scramble sequence served as a control. Both cell groups were subsequently exposed to 5 µM MeHg for 6 h **A** Representative western blot image of phospho-JAK2 (Y1007/1008), total JAK2, phospho-STAT3 (Y705), total STAT3, SOCS3, and β-actin. **B** Densitometry quantification of the phospho-JAK2/JAK2 ratio (*n* = 7 independent cell culture preparations). **C** Densitometry quantification of the phospho-STAT3/STAT3 ratio (*n* = 7 independent cell culture preparations). **D** Densitometry quantification of SOCS3 protein levels (*n* = 7). **E** Representative western blot images of Nrf2, KEAP1, SOD2, and β-actin. **F** Densitometry quantification of Nrf2 protein levels (*n* = 7 independent cell culture preparations). **G** Densitometry quantification of KEAP1 protein levels (*n* = 7 independent cell culture preparations). **H** Densitometry quantification of SOD2 protein levels (*n* = 7 independent cell culture preparations). **I** Representative western blot images of HO-1 and β-actin. **J** Densitometry quantification of HO-1 protein levels (*n* = 7 independent cell culture preparations). **K** qPCR analysis of *Stat3* mRNA levels (*n* = 6 independent cell culture preparations). **L** qPCR analysis of *Socs3* mRNA levels (*n* = 6 independent cell culture preparations). **M** qPCR analysis of *Il-6* mRNA levels (*n* = 6 independent cell culture preparations). **N** qPCR analysis of *Nfe2l2* mRNA levels (*n* = 6 independent cell culture preparations). **O** qPCR analysis of *Keap1* mRNA levels (*n* = 6 independent cell culture preparations). **P** qPCR analysis of *Sod2* mRNA levels (*n* = 6 independent cell culture preparations). **Q** qPCR analysis of *Hmox1* mRNA levels (*n* = 6 independent cell culture preparations). **R** qPCR analysis of *Nqo1* mRNA levels (*n* = 6 independent cell culture preparations). **S** qPCR analysis of *Slc7a11* mRNA levels (*n* = 6 independent cell culture preparations). Data are expressed as mean ± SD. * indicates *p* < 0.05

**Fig. 4:**
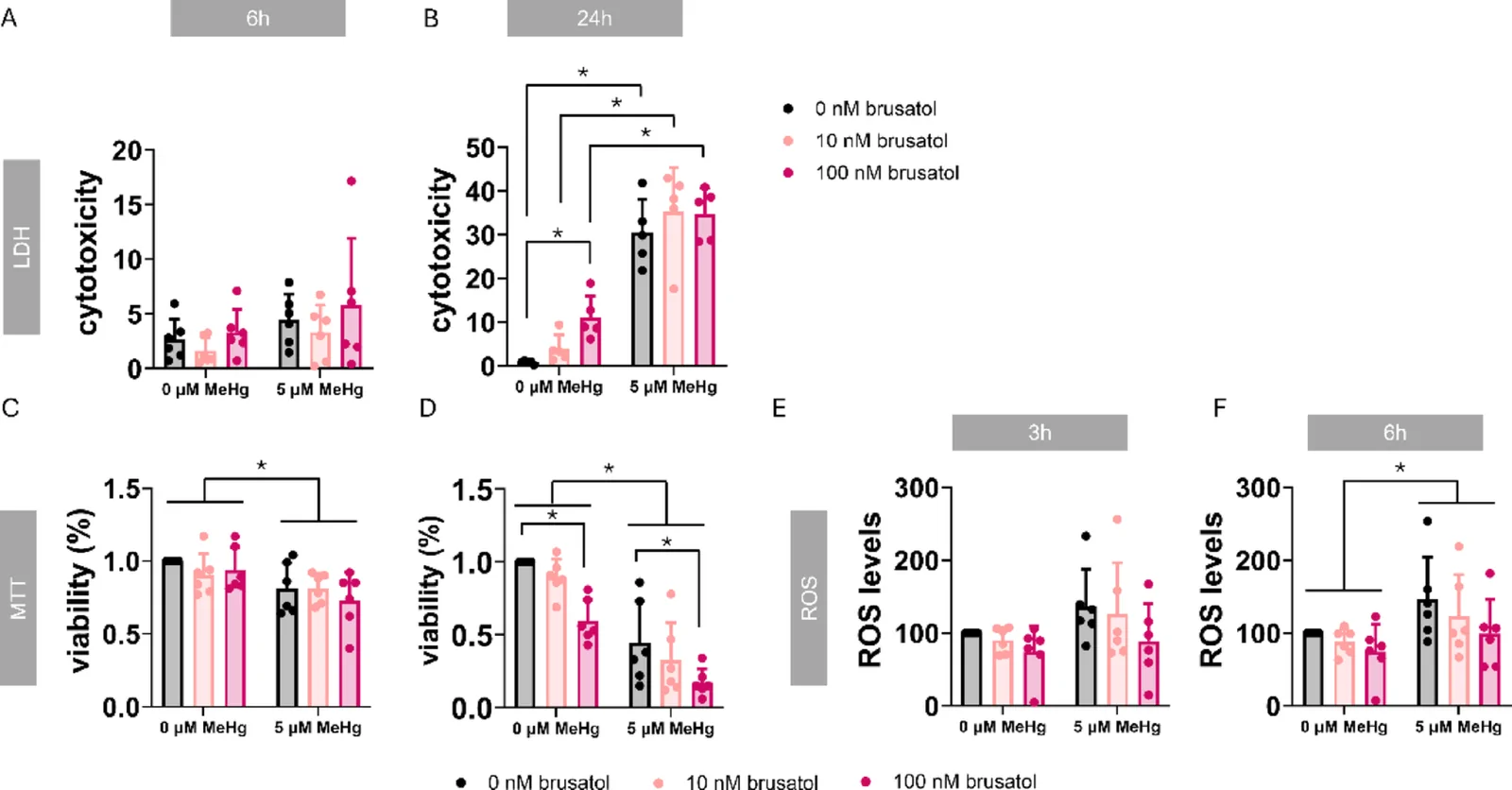
Inhibition of Nrf2 by brusatol causes lethality without affecting ROS production in mouse hypothalamic neuronal GT1-7 cells. **A** Cytotoxicity measured by LDH release in GT1-7 cells pre-treated 1 h with 10 or 100 nM brusatol, followed co-exposure to 5 µM MeHg for 6 h (*n* = 6 independent cell culture preparations). **B** Cytotoxicity measured by LDH release in GT1-7 cells pre-treated 1 h with 10 or 100 nM brusatol, followed co-exposure to 5 µM MeHg for 24 h (*n* = 5 independent cell culture preparations). **C** Cell viability assessed by MTT assay in GT1-7 cells pre-treated 1 h with 10 or 100 nM brusatol, followed co-exposure to 5 µM MeHg for 6 h (*n* = 6 independent cell culture preparations). **D** Cell viability assessed by MTT assay in GT1-7 cells pre-treated 1 h with 10 or 100 nM brusatol, followed co-exposure to 5 µM MeHg for 24 h (*n* = 6 independent cell culture preparations). **E** ROS production measured using the CM-H_2_DCFDA probe in GT1-7 cells pre-treated 1 h with 10 or 100 nM brusatol, followed co-exposure to 5 µM MeHg for 3 h (*n* = 6 independent cell culture preparations). **F** ROS production measured using the CM-H_2_DCFDA probe in GT1-7 cells pre-treated 1 h with 10 or 100 nM BT, followed co-exposure to 5 µM MeHg for 6 h (*n* = 6 independent cell culture preparations). Data are expressed as mean ± SD. * indicates *p* < 0.05

**Fig. 5:**
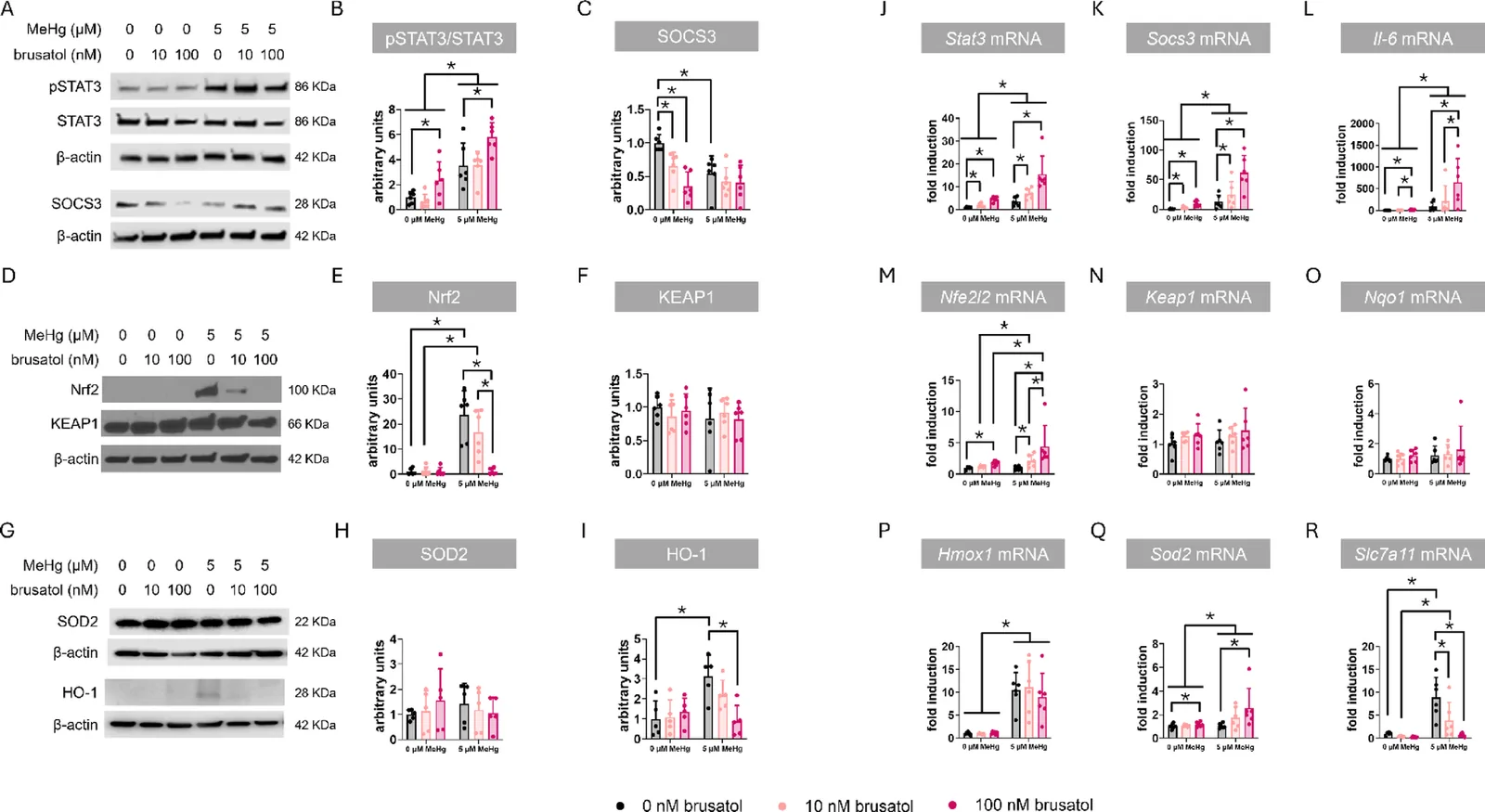
Inhibition of Nrf2 by brusatol enhances MeHg-induced STAT3 activity in mouse hypothalamic neuronal GT1-7 cells. GT1-7 cells were pre-treated 1 h with 10 or 100 nM brusatol, followed by co-exposure to 5 µM MeHg for 6 h. **A** Representative western blot images of phospho-STAT3 (Y705) total STAT3, and β-actin, and SOCS3 and β-actin. **B** Densitometry quantification of phospho-STAT3/STAT3 (*n* = 6 independent cell culture preparations). **C** Densitometry quantification of SOCS3 protein levels (*n* = 6 independent cell culture preparations). **D** Representative western blot images of Nrf2, KEAP1, and β-actin. **E** Densitometry quantification of Nrf2 protein levels (*n* = 6 independent cell culture preparations). **F** Densitometry quantification of KEAP1 protein levels (*n* = 6 independent cell culture preparations). **G** Representative western blot images of SOD2 and β-actin, and HO-1, and β-actin. **H** Densitometry quantification of SOD2 protein levels (*n* = 5 independent cell culture preparations). **I** Densitometry quantification of HO-1 protein levels (*n* = 5 independent cell culture preparations). **J** qPCR analysis of *Stat3* mRNA levels (*n* = 6 independent cell culture preparations). **K** qPCR analysis of *Socs3* mRNA levels (*n* = 6 independent cell culture preparations). **L** qPCR analysis of *Il-6* mRNA levels (*n* = 6 independent cell culture preparations). **M** qPCR analysis of *Nfe2l2* mRNA levels (*n* = 6 independent cell culture preparations). **N** qPCR analysis of *Keap1* mRNA levels (*n* = 6 independent cell culture preparations). **O** qPCR analysis of *Nqo1* mRNA levels (*n* = 6 independent cell culture preparations). **P** qPCR analysis of *Hmox1* mRNA levels (*n* = 6 independent cell culture preparations). **Q** qPCR analysis of *Sod2* mRNA levels (*n* = 6 independent cell culture preparations). **R** qPCR analysis of Slc7a11 mRNA levels (*n* = 6 independent cell culture preparations). Data are expressed as mean ± SD. * indicates *p* < 0.05

**Fig. 6:**
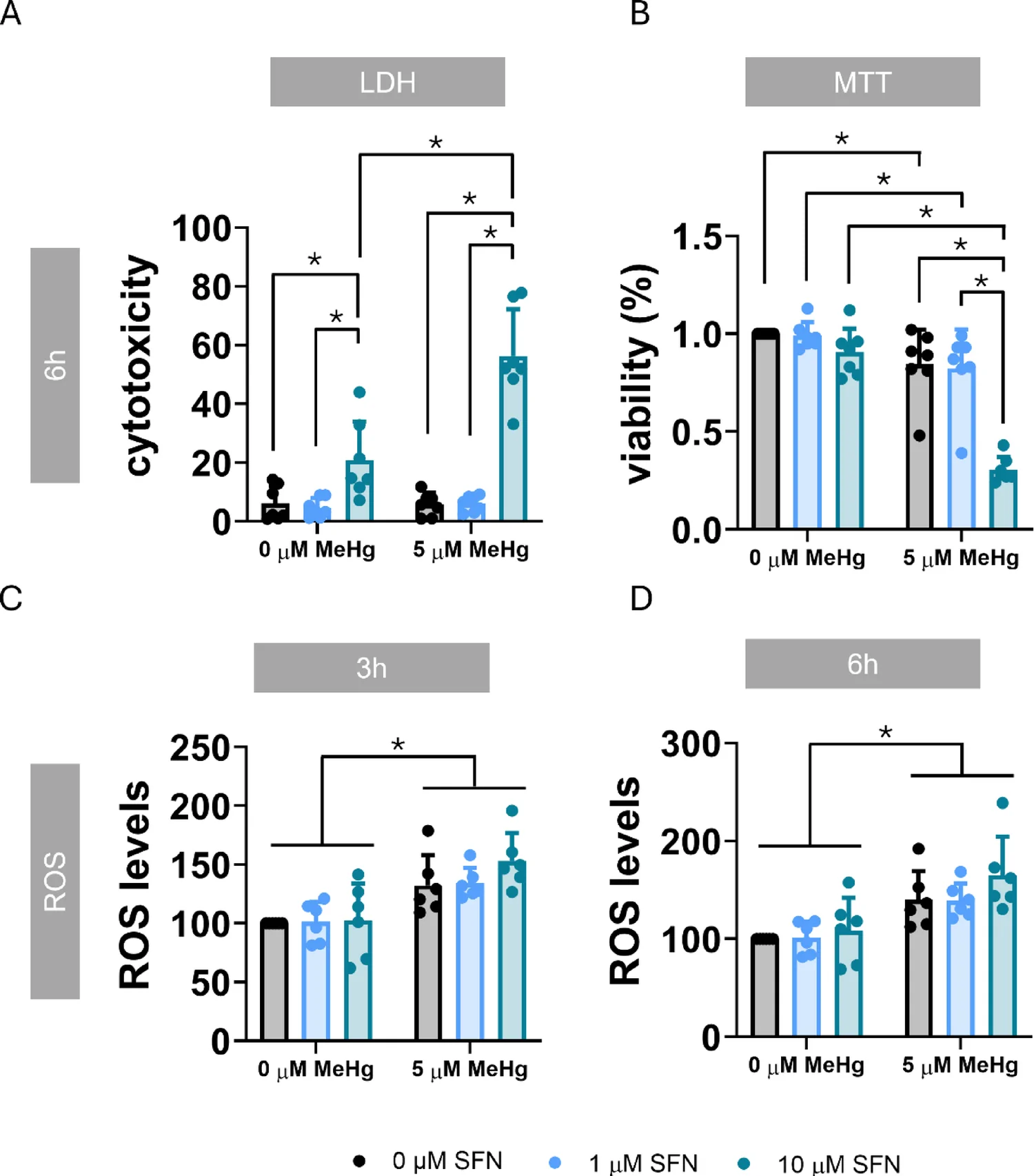
Activation of Nrf2 by sulforaphane (SFN) does not ameliorate MeHg-induced ROS production in mouse hypothalamic neuronal GT1-7 cells. **A** Cytotoxicity measured by LDH release in GT1-7 cells pre-treated 30 min with 1 or 10 µM SFN, followed by co-exposure to 5 µM MeHg for 6 h (*n* = 7 independent cell culture preparations). **B** Cell viability assessed by MTT assay in GT1-7 cells pre-treated 30 min with 1 or 10 µM SFN, followed by co-exposure to 5 µM MeHg for 6 h (*n* = 7 independent cell culture preparations). **C** ROS production measured using the CM-H_2_DCFDA probe in GT1-7 cells pre-treated 30 min with 1 or 10 µM SFN, followed by co-exposure to 5 µM MeHg for 3 h (*n* = 6 independent cell culture preparations). **D** ROS production measured using the CM-H_2_DCFDA probe in GT1-7 cells pre-treated 30 min with 1 or 10 µM SFN, followed by co-exposure to 5 µM MeHg for 6 h (*n* = 6 independent cell culture preparations). Data are expressed as mean ± SD. * indicates *p* < 0.05

**Fig. 7:**
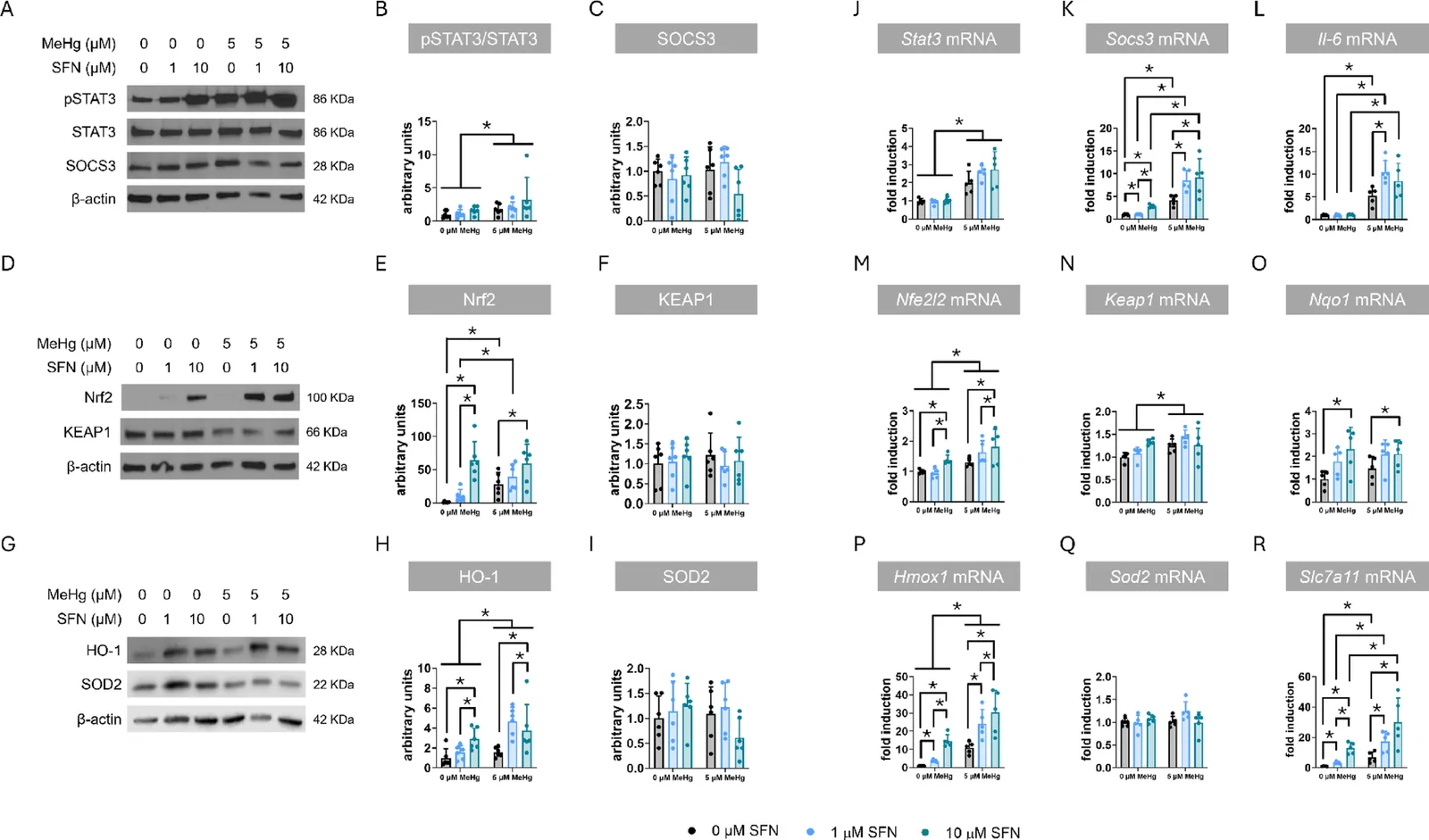
Activation of Nrf2 by sulforaphane (SFN) does not enhance MeHg-Induced STAT3 signaling in mouse hypothalamic neuronal GT1-7 cells. GT1-7 cells were pre-treated 30 min with 1 or 10 µM SFN followed by 6 h co-exposure to 5 µM MeHg. **A** Representative western blot images of phospho-STAT3 (Y705), total STAT3, SOCS3, and β-actin. **B** Densitometry quantification of phospho-STAT3/STAT3 ratio (*n* = 6 independent cell culture preparations). **C** Densitometry quantification of SOCS3 protein levels (*n* = 6 independent cell culture preparations). **D** Representative western blot images of Nrf2, KEAP1, and β-actin. **E** Densitometry quantification of Nrf2 protein levels (*n* = 6 independent cell culture preparations). **F** Densitometry quantification of KEAP1 protein levels (*n* = 6 independent cell culture preparations). **G** Representative western blot images of SOD2, HO-1, and β-actin. H) Densitometry quantification of SOD2 protein levels (*n* = 6 independent cell culture preparations). **I** Densitometry quantification of HO-1 protein levels (*n* = 6 independent cell culture preparations). **J** qPCR analysis of *Stat3* mRNA levels (*n* = 5 independent cell culture preparations). **K** qPCR analysis of *Socs3* mRNA levels (*n* = 5 independent cell culture preparations). **L** qPCR analysis of *Il-6* mRNA levels (*n* = 5 independent cell culture preparations). **M** qPCR analysis of *Nfe2l2* mRNA levels (*n* = 5 independent cell culture preparations). **N** qPCR analysis of *Keap1* mRNA levels (*n* = 5 independent cell culture preparations). **O** qPCR analysis of *Nqo1* mRNA levels (*n* = 5 independent cell culture preparations). **P** qPCR analysis of *Hmox1* mRNA levels (*n* = 5 independent cell culture preparations). **Q** qPCR analysis of *Sod2* mRNA levels (*n* = 5 independent cell culture preparations). **R** qPCR analysis of *Slc7a11* mRNA levels (*n* = 5 independent cell culture preparations). Data are expressed as mean ± SD. * indicates *p* < 0.05

Cells were sub-cultured into different plates and exposed with 5 µM methylmercury (II) chloride (MeHg) (Sigma-Aldrich, cat. no. 442534) for various durations depending on the assay. Time-course experiments for gene and protein expression were conducted at 0, 1, 3, and 6 h (Fig. 1). For all the experiments involving STAT3 depletion (pharmacological inhibition or genetic ablation) (Figs. 2 and 3), and for protein and gene expression studies in combination with brusatol (Fig. 5) or Sulforaphane (SFN) (Fig. 7), MeHg exposure was carried out for 6 h. For cell viability experiments, exposure durations were 3, 6–24 h, whereas ROS production was assessed after 3–6 h of MeHg exposure (Figs. 4 and 6).

MeHg concentration was selected based on our previous studies [41, 48] and literature reports [49], and it is expected to result in a maximal accumulation of 50 µHg/g protein in the brain. This concentration is considered relevant to both human studies and real-life exposure scenarios, and is expected to produce accumulations comparable to those reported to cause sub-toxic threshold effects in mammalian CNS [50].

### STAT3 Inhibitor Treatment

To investigate the role of STAT3 in Nrf2 induction, cells were pre-treated for 1 h with either 10 or 100 µM AG490 (Tyrphostin B42, Santa Cruz Biotechnology, Santa Cruz biotechnology, Inc. cat. no. sc-202046) or with 15 or 30 µM of STAT3 Inhibitor XIII, C188-9 (CalbioChem, cat. no. 573128). This pre-treatment was followed by 6 h of co-treatment with 5 µM MeHg.

### Brusatol Treatment

To investigate the role of Nrf2 in STAT3 activation, cells were pre-treated 1 h with either 10 or 100 nM brusatol (Sigma-Aldrich, cat. no. SML1868), a plant-derived quassinoid which promotes Nrf2 ubiquitination and degradation. This pre-treatment was followed by 6 h of co-treatment with 5 µM MeHg.

### L-Sulforaphane (SFN) Treatment

To fully understand the role of Nrf2 in STAT3 phosphorylation, cells were pre-treated for 30 min with 1 or 10 µM L-Sulforaphane (SFN, Sigma-Aldrich, cat. no. S6317), a cruciferous vegetable-derived organosulfur compound that induces Nrf2-mediated signaling. This pre-treatment was followed by 6 h of co-treatment with 5 µM MeHg. For dose-response experiments, SFN was tested at 1, 2, 5, and 10 µM for LDH and MTT assays, and at 2, 5, and 10 µM for Western Blot analysis.

### Cytotoxicity and Cell Viability Measurements

Cytotoxicity and cell viability assays were performed in cells seeded in a 96-well plate. The cytotoxic effect of the different compounds was measured by lactate dehydrogenase (LDH) release using the CyQUANT LDH Cytotoxicity Assay Kit (Thermo Fisher Scientific, cat. no. C20301), following the manufacturer’s instructions. Briefly, after exposure to the compounds at the concentrations and durations specified in the figure legends, cell culture supernatants were transferred to a new 96-wells plate and incubated with the substrate mix 30 min at room temperature. After stopping the reaction with the provided buffer, absorbance was measured at 490 nm and 680 nm.

Cell viability was assessed with MTT (3-(4,5-dimethylthiazol-2-yl)−2,5-diphenyltetrazolium bromide) (Thermo Fisher Scientific, cat. no. M694). Following exposure to the compounds at the concentrations and durations specified in the figure legends, the culture medium was removed, and the cells were incubated with 0.5 µg/µL MTT in Hank’s balanced salt solution (HBSS) (Gibco™, cat. no. 14175095) for 2 h at 37 °C in a humidified atmosphere containing 5% CO_2_. Subsequently, the supernatant was removed, and dimethyl sulfoxide (DMSO, Sigma Aldrich, cat. no. D8418) was added to solubilize the formazan crystals formed by viable cells through the reduction of MTT. The absorbance was measured at 540 nm with a microplate reader.

### Measurement of Reactive Oxygen Species (ROS) Production

Intracellular ROS production was assessed using cell-permeant fluorescent probe CM-H_2_DCFDA (5-(and-6) chloromethyl-2′7′-dichloro dihydrofluorescein diacetate acetyl ester, Thermo Fisher Scientific, cat. no. C6827), a sensitive indicator of oxidative stress. Briefly, cells were seeded in black 96 wells plate. The cells were then incubated with 5 µM CM-H_2_DCFDA diluted in HBSS 30 min at 37 °C in a humidified atmosphere containing 5% CO_2_ in a tissue culture incubator. After incubation with the probe, cells were exposed to the different compounds at the times and concentrations indicated in the figure legends. Fluorescence intensity, indicative of intracellular ROS levels, was then measured using microplate reader with excitation and emission wavelengths of 492 nm and 525 nm, respectively.

### Western Blot Analysis

Whole cell protein extracts were obtained by lysing cells with ice-cold radioimmunoprecipitation assay (RIPA) buffer (Sigma Aldrich, cat. no. R0278) supplemented with protease inhibitor cocktails 2 and 3 (Sigma Aldrich, P5726 and P0044, respectively) and Halt™ phosphatase inhibitor cocktail (Thermo Fisher Scientific, cat. no. 78437). The lysates were then sonicated and centrifuged at 10.000 g for 10 min at 4 °C. The protein concentration of the supernatant was determined using the Pierce BCA Protein Assay Kit (Thermo Fisher Scientific, cat. no. 23225).

Nuclear and cytoplasmic subcellular fractions were isolated using the NE-PER Nuclear and Cytoplasmic Extraction Reagent Kit (Thermo Fisher Scientific, cat. no. 78833), according to the manufacturer’s protocol.

Samples were diluted in Laemmli sample buffer (Bio-Rad, cat. no. 1610737) and boiled at 100 °C for 5 min. Then, 10 µg of protein were resolved by 4–20% sodium dodecyl sulfate-polyacrylamide gel (SDS-PAGE, Bio-Rad cat. no. 4561096) and subsequently transferred to nitrocellulose membranes. Membranes were blocked with 5% bovine serum albumin (BSA, Sigma-Aldrich, cat. no. A3059) diluted in Tris-buffer saline with 0.1% Tween-20 (TBS-T) for 1 h at room temperature. The membranes were then incubated overnight at 4 °C with primary antibodies diluted in 5% BSA in TBS-T buffer. The next day, after three washes with TBS-T, membranes were incubated with appropriate horseradish peroxidase-conjugated secondary antibody. Protein bands were visualized using a chemiluminescent reagent (Thermo Fisher Scientific cat. no. 34579 or cat. no. 37071). Band densitometry was quantified using ImageJ software (NIH) [51]. For whole cell lysates densitometry was normalized to β-actin (Sigma-Aldrich, cat. no. A1978). Glyceraldehyde 3-phosphate dehydrogenase (GAPDH) (Cell Signaling Technology, cat. no. 5174) and TATA-binding protein (TBP) (Cell Signaling Technology, cat. no. 44059) served as loading/enrichment controls for cytoplasmic and nuclear fractions, respectively.

The following antibodies were used: rabbit anti-phospho-STAT3 (Tyr705) (Cell Signaling Technologies, cat. no. 9145), rabbit anti-STAT3 (Cell Signaling Technology, cat. no. 12640), rabbit anti-SOCS3 (Cell Signaling Technology, cat. no. 2932), rabbit anti-Nrf2 (Cell Signaling Technology, cat. no. 12721), mouse anti-KEAP1 (R&D Systems, cat. no. MAB3024), rabbit anti-phospho-JAK2 (Tyr1007/1008) (Thermo Fisher Scientific, cat. no. 44-426G), rabbit anti-JAK2 (Cell Signaling Technology, cat. no. 3230), rabbit anti-SOD2 (Cell Signaling Technology, cat. no. 13141), and goat anti-HO-1 (R&D Systems, cat. no. AF3169).

### Quantitative Real Time PCR

Total RNA was isolated from cell cultures using the RNeasy Mini kit (Qiagen, 74104) according to the manufacturer’s instructions. RNA concentration was measured with a Nanodrop 2000 Spectrophotometer (Thermo Fisher Scientific, cat. no. ND2000). Next, cDNA was synthesized using the High Capacity cDNA Reverse Transcription kit (Applied Biosystems, cat. no. 4368814) according to the manufacturer’s instructions. Gene expression levels were quantified by qRT-PCR using TaqMan Gene Expression assay (Applied Biosystems) probes: *Nfe2l2* (assay ID: Mm00477784_m1;), *Keap1* (assay ID: Rn01448220_m1;); *Stat3* (assay ID: Mm01219775_m1); *Socs3* (assay ID: Mm00545913_s1); *Il-6* (assay ID: Mm00446190_m1); *Hmox1* (assay ID: Mm00516005_m1), *Slc7a*11 (assay ID: Mm00442530_m1), *Sod2* (assay ID: Mm01313000_m1), and *Nqo1* (assay ID: Rn00566528_m1). Gene expression levels were determined using the 2^−ΔΔCt^ method [52], and the data were normalized to *Gapdh* (Assay ID: Mm99999915_g1), which served as a housekeeping gene.

### Statistical Analysis

Statistical analyses were performed using the Statistical Package for Social Sciences (SPSS) version 29.0.2.0(20) software. GraphPad Prism 10.4.1 software was used for graphical data representation. No sample size calculations were performed; sample sizes for each experiment were determined based on similar previous published studies [49, 53]. To determine if the data were normally distributed, we applied the Shapiro-Wilk and Kolmogorov-Smirnov tests. No test for outliers was conducted. All statistical tests were conducted as two-tailed tests.

The effects of MeHg at different time points were analyzed by one-way analysis of variance (ANOVA) followed by Bonferroni’s post-hoc test. When normality was not met, we used the non-parametric Kruskal-Wallis test, adjusted by Bonferroni correction.

The effects of STAT3 inhibitors and STAT3 knockdown on MeHg-induced responses were determined by two-way ANOVA with MeHg exposure and inhibitor/shRNA treatment as main factors, followed by Bonferroni’s post-hoc test. F values were indicated as: F_MeHg_ (Methylmercury significant effect), F_AG_ (AG490 significant effect), F_C_ (C188-9 significant effect), F_KO_ (STAT3 knockdown significant effect), F_IntM−A_ (Methylmercury x AG490 interaction significant effect), F_IntM−C_ (Methylmercury x C188-9 interaction significant effect), and F_IntM−K_ (Methylmercury x STAT3 knockdown significant effect). We applied logarithmic or square root transformation to data that did not meet normality assumptions, aiming for a closer approximation to a normal distribution.

Similarly, the effects of brusatol and SFN on MeHg-induced responses were analyzed using two-way ANOVA with MeHg exposure and compound (brusatol or SFN) treatment as main factors, followed by Bonferroni’s post-hoc test. F values were indicated as: F_MeHg_ (Methylmercury significant effect), F_BT_ (brusatol significant effect), F_SFN_ (Sulforaphane significant effect), F_InfM−B_ (Methylmercury x brusatol interaction significant effect), and F_IntM−S_ (Methylmercury x Sulforaphane significant effect). To achieve a more normal distribution for data that failed the normality test, we performed either a logarithmic or square root transformation.

All data are presented as mean ± standard deviation (SD), and statistical significance was defined as *p* < 0.05.

## Results

### MeHg Induced Nrf2 Protein Expression in a Time-Dependent Manner, Concomitant With STAT3 Activation in GT1-7 Cells

To investigate the induction of NRF2 by MeHg in GT1-7 cells, we examined changes in Nrf2 protein levels and *Nfe2l2* gene expression in culture cells exposed to 5 µM MeHg at different times. Western blot analysis revealed a significant time-dependent increase in NRF2 protein levels (F_(3,20)_ = 4.277, *p* = 0.017), with the most pronounced effect observed at 6 h post-exposure (*p* = 0.037 compared with time 0 h). (Fig. 1A and B). In contrast, no significant alterations in *Nfe2l2* mRNA expression were detected following MeHg exposure (Fig. 1E).

KEAP1, the endogenous repressor of Nrf2, typically targets Nrf2 for ubiquitination and subsequent proteasomal degradation under physiological conditions. In response to oxidative stress, Nrf2 dissociates from KEAP1, translocates to the nucleus, and induces the expression of phase II detoxifying enzymes [54]. Here, no significant changes were observed in KEAP1 protein levels and *Keap1* gene expression following MeHg exposure (Fig. 1A, C, and F).

As previously reported [41], MeHg induced STAT3 phosphorylation in a time-dependent manner (H_(3)_ = 19.700, *p* < 0.001) (Fig. 1A and D), with a significant increase observed at 6 h (*p* < 0.001 compared with time 0 h). Our findings indicate that 5 µM MeHg can simultaneously activate both the Nrf2 and STAT3 pathways in hypothalamic neuronal GT 1–7 cells.

### STAT3 Impairment Exacerbated Nrf2 Activation in Response to MeHg Exposure

STAT3 inhibition exacerbated the activation of antioxidant defense in GT1-7 cells exposed to MeHg, increasing the expression of Nrf2 antioxidant downstream genes [41]. These findings prompted us to investigate the relationship between STAT3 and Nrf2. Specifically, we examined whether pharmacological inhibition of STAT3 was associated with increased Nrf2 abundance in the nuclear fraction. To achieve this aim, we inhibited STAT3 phosphorylation by targeting the upstream protein kinase JAK2 using AG490 [55], or by directly inhibited STAT3 activity using C188-9 [56].

As expected, MeHg increased the Nrf2 signal detected in the nuclear fraction (F_MeHg(1,48)_ = 8.772, *p* = 0.005) (Fig. 2A). However, due to the low cytoplasmic Nrf2 signal, we cannot rule out the contribution of other mechanisms such as increased nuclear protein stabilization or local transition. Thus, while our data are consistent with increased Nrf2 nuclear accumulation, they do not demonstrate active translocation. When STAT3 phosphorylation was pharmacologically inhibited, Nrf2 protein levels in the nucleus increased significantly (F_AG−C(2,48)_ = 34.252, *p* < 0.001) (Fig. 2A). Interestingly, a significant interaction was found, showing that inhibition of the JAK2/STAT3 signaling pathway with 100 µM AG490 induced *Nfe2l2* mRNA expression in cells exposed to MeHg (F_IntM−A(2,36)_ = 5.217, *p* = 0.010) (Fig. 2B). A similar effect was observed when STAT3 phosphorylation was directly inhibited by 30 µM C188-9 (significant interaction between MeHg and C188-9: F_IntM−C(2,36)_ = 7.680, *p* = 0.002) (Fig. 2F).

Pharmacological inhibition of JAK2 phosphorylation and STAT3 activity led to diverse effects on *Keap1* gene expression. A high concentration of AG490 (100 µM) decreased *Keap1* mRNA levels (F_AG(2,36)_ = 33.456, *p* < 0.001) (Fig. 2C). In contrast, C188-9 had no effect on *Keap1* gene expression (Fig. 2G). These findings suggest that JAK2 may regulate *Keap1* gene expression through a mechanism independent of STAT3.

Next, we assessed the gene expression of Nrf2 target genes, Solute Carrier Family 7 Member 11 (SLC7A11) and NAD(P)H quinone oxidoreductase 1 (NQO1). SLC7A11 is a component of the xCT system, a heteromeric, sodium-independent, anionic amino acid transport system, and its expression is also regulated by STAT3 [18]. AG490 significantly exacerbated MeHg-induced expression of *Slc7a11* mRNA, as shown by the significant interaction (F_IntM−A(2,36)_ = 3.704, *p* = 0.034) (Fig. 2D). However, AG490 failed to induce *Nqo1* mRNA expression (Fig. 2E). Similarly, when STAT3 phosphorylation was inhibited with C188-9, Slc*7a11* gene expression increased significantly (F_C(2,35)_ = 48.705, *p* < 0.001) (Fig. 2H), while no changes were observed in *Nqo1* gene expression (Fig. 2I). Notably, neither inhibition of the pathway with AG490 nor C188-9 affected *Nqo1* mRNA expression, suggesting that JAK2/STAT3 may not be involved in its regulation.

Given the observed differences between the two STAT3 inhibitors, and to better understand the relationship between STAT3 and Nrf2 signaling pathways, we transfected cells with lentivirus carrying either STAT3 shRNA or a scramble sequence. Western blot analysis confirmed that STAT3 protein levels were downregulated by the lentivirus carrying STAT3 shRNA (Fig. 3Aand C). A significant interaction between MeHg and STAT3 shRNA (F_IntM−K(1,24)_ = 75.053, *p* < 0.001) showed that MeHg induced STAT3 activation only in cells transfected with scramble shRNA(*p* < 0.001) (Fig. 3A, C). Upstream of STAT3, STAT3 deficiency has no effect on pJAK2/JAK2 protein levels, while MeHg induced JAK2 activity (F_MeHg(1,24)_ = 8.109, *p* = 0.009) (Fig. 3A and B). In contrast, neither MeHg nor STAT3 deficiency affected the protein levels of suppressor of cytokine signaling 3 (SOCS3) (Fig. 3A and D), a protein whose expression is induced by the same JAK2/STAT3 pathway and mediates the inactivation of the pathway, generating a negative feedback loop [57].

Both MeHg (F_MeHg(1,24)_ = 29.687, *p* < 0.001) and STAT3 deletion (F_KO(1,24)_ = 5.309, *p* = 0.030) increased Nrf2 expression in cells exposed to MeHg (Fig. 3E, F). However, STAT3 knockdown did not affect KEAP1 protein levels (Fig. 3E and G). STAT3 has been shown to be recruited to the SOD2 promoter, upregulating its expression [17]. However, in our experiments, we did not observe any significant effect of STAT3 knockout on SOD2 protein levels (Fig. 3E, H). Additionally, we analyzed changes in HO-1 protein levels. MeHg significantly induced the expression of HO-1, an effect that was further exacerbated by STAT3 deletion (F_IntM−K(1,24)_ = 5.184, *p* = 0.032) (Fig. 3I, J).

To further investigate the relationship between Nrf2 and STAT3, we performed qPCR analysis to examine the expression of several downstream genes of these transcription factors. First, we confirmed the successful knockdown of *Stat3* mRNA by shSTAT3 (Fig. 3K). Additionally, a significant interaction between MeHg and STAT3 shRNA (F_IntM−K(1,20)_ = 15.632, *p* < 0.001), showed that MeHg induced *Stat3* gene expression in scramble-transfected cells (*p* < 0.001) (Fig. 3K). Similarly, MeHg induced *Socs3* mRNA expression, an effect that was blocked by STAT3 downregulation (F_IntM−K(1,20)_ = 36.115, *p* < 0.001) (Fig. 3L). Next, we examined, *Il-6* mRNA expression, a known STAT3 target gene. While MeHg exposure induced *Il-6* mRNA expression in STAT3 deficient cells, the response was significantly attenuated compared to wild-type cells (F_IntM−K(1,20)_ = 9.805, *p* = 0.005) (Fig. 3M), indicating that STAT3 contributes to the induction of IL-6 in response to MeHg.

Next, we investigated the gene expression of *Nfe2l2*, the gene that encoding Nrf2, and *Keap1*. MeHg exposure significantly induced *Nfe2l2* mRNA expression (F_MeHg(1,20)_ = 9.545, *p* = 0.006), with no observed effect of STAT3 deletion (Fig. 3N). In contrast, neither STAT3 deficiency nor MeHg exposure affected *Keap1* mRNA expression (Fig. 3O), which was consistent with the results obtained when STAT3 activity was pharmacologically inhibited with a small-molecular inhibitor of STAT3 C188-9 (Fig. 2G).

We further assessed changes in the expression in Nrf2 downstream genes. MeHg induced *Nqo1* gene expression (F_MeHg(1,20)_ = 13.084 *p* = 0.002), an effect which was exacerbated by STAT3 deletion (F_KO(1,20)_ = 29.661, *p* < 0.001) (Fig. 3R). Similarly, STAT3 downregulation further enhanced MeHg-induced *Hmox1* gene expression, as indicated by a significant interaction between MeHg and STAT3 shRNA (F_IntM−K(1,20)_ = 33.458, *p* < 0.001) (Fig. 3Q). Together with the increase in HO-1 protein levels observed after STAT3 deletion, these downstream molecular changes support the interpretation that STAT3 impairment enhances an Nrf2-driven antioxidant response in MeHg-exposed GT1-7 cells.

Finally, we analyzed the gene expression of two genes, *Sod2* and *Slc7a11*, which are known targets of both Nrf2 and STAT3. While STAT3 depletion has no effects on *Sod2* or *Slc7a11* mRNA expression (Fig. 3P and S), MeHg exposure significantly induced *Slc7a11* gene expression (F_MeHg(1,20)_ = 140.847, *p* < 0.001) (Fig. 3S).

### Nrf2 Pharmacological Inhibition Exacerbated STAT3 Activation in Response to MeHg Exposure

Our next aim was to determine whether Nrf2 modulates STAT3 activation. To this end, we pharmacologically inhibited MeHg-induced Nrf2 pathway using brusatol, a plant extract from *Brucea javanica*, which inhibits Nrf2 activity by promoting its ubiquitination and degradation [58]. First, we evaluated the cytotoxic effects of brusatol in presence and absence of MeHg. GT1-7 cells were pre-treated with various doses of brusatol for 1 h followed by co-exposure to MeHg. No significant cytotoxicity was detected at 6 h of MeHg exposure, as assessed by LDH assay (Fig. 4A). However, the MTT assay revealed a significant decrease in cell viability induced by MeHg at 6 h (F_MeHg(1,30)_ = 11.622, *p* = 0.002) (Fig. 4C). At 24 h, MeHg-induced cytotoxicity was enhanced by brusatol (F_IntM−B(2,24)_ = 5.550, *p* = 0.010) (Fig. 4B). The cytotoxic effect of MeHg (F_MeHg(1,30)_ = 73.878, *p* < 0.001) and high-dose brusatol (F_BT(2,30)_ = 11.038, *p* < 0.001, post hoc *p* < 0.001 compared to control) at 24 h were observed in the MTT assay (Fig. 4D).

Next, we examined the impact of brusatol on ROS production. While 5 µM MeHg induced ROS levels, the increase was statistically significant only at 6 h (F_MeHg(1,30)_ = 6.293, *p* = 0.018). Notably, brusatol did not further influence ROS production (Fig. 4E, F). The lack of exacerbation in ROS production in brusatol-treated cells suggests that antioxidant defenses against MeHg-induced changes in redox status may also involve mechanisms independent of Nrf2.

Brusatol treatment induced STAT3 phosphorylation in GT1-7 cells (F_BT(2,30)_ = 10.106, *p* < 0.001) (Fig. 5A and B). Notably, a significant interaction was found for SOCS3 protein expression (F_IntM−B(2,30)_ = 4.081, *p* = 0.027), showing that brusatol decreased SOCS3 protein expression, an effect reversed in cells exposed to MeHg (Fig. 5 A, C). This suggests that the increase in the pSTAT3/STAT3 ratio may, at least in part, be mediated by a decrease in SOCS3. As expected, brusatol inhibited MeHg-induced Nrf2 protein levels (F_IntM−B(2,30)_ = 6.423, *p* = 0.001) (Fig. 5D and E). Additionally, the lack of changes in KEAP1 protein levels supports the idea that brusatol-mediated Nrf2 inhibition occurs through mechanisms independent of KEAP1 (Fig. 5D, F).

To further corroborate these findings, we analyzed protein expression of SOD2 and HO-1, which are regulated by Nrf2. Neither MeHg nor brusatol affected SOD2 protein levels (Fig. 5G, H). However, HO-1 protein levels correlated with the changes in Nrf2; MeHg increased its expression, an effect that was abrogated by brusatol, as shown by the significant interaction between MeHg and brusatol (F_IntM−B(2,24)_ = 6.035, *p* = 0.008) (Fig. 5G and I).

To determine whether the transcriptional activity of STAT3 and Nrf2 reflected the changes observed in their protein levels we evaluated gene expression changes using qPCR. The mRNA levels of *Stat3* correlated with the changes observed for the pSTAT3/STAT3 ratio. MeHg induced *Stat3 gene expression* (F_MeHg(1,30)_ = 91.727, *p* < 0.001), which was further increase by brusatol (F_BT(2,30)_ = 49.417, *p* < 0.001) (Fig. 5J). Next, we examined the changes in STAT3 target genes, *Socs3*, and *Il-6*. Similar to *Stat3* m RNA, MeHg induced *Socs3* gene expression (F_MeHg(1,30)_ = 57.630, *p* < 0.001) that was exacerbated by brusatol treatment (F_BT(2,30)_ = 20.572, *p* < 0.001) (Fig. 5K). Similarly, we detected a significant effect of MeHg (F_MeHg(1,30)_ = 41.264, *p* < 0.001) and brusatol treatment on *Il-6* mRNA expression (F_BT(2,30)_ = 9.184, *p* < 0.001) (Fig. 5L).

Next, we assessed changes in *Nfe2l2* and *Keap1* mRNA expression. A significant interaction (F_IntM−B(2,30)_ = 3.539, *p* = 0.042) showed that MeHg induced *Nfe2l2* gene expression and this induction was further increased by brusatol treatment (Fig. 5M). The effects of brusatol on *Nfe2l2* gene expression may represent a compensatory mechanism to counteract the increase in Nrf2 degradation. The absence of changes in *Keap1* gene expression (Fig. 5N), along with no changes in protein levels, suggests that the effects of brusatol on Nrf2 may be independent of KEAP1. To further characterize the effects of brusatol on Nrf2 activity, we evaluated the expression of several Nrf2-induced antioxidant genes. While no changes were detected in *Nqo1* gene expression (Fig. 5O), MeHg induced *Hmox1* mRNA expression (F_MeHg(1,30)_ = 198.890, *p* < 0.001) (Fig. 5P). Although brusatol inhibited Nrf2 protein levels, no significant effects were observed for either *Nqo1* (Fig. 5O) or *Hmox1* (Fig. 5P) gene expression. These findings suggest that alternative mechanisms may be involved in MeHg-induced regulation of antioxidant gene expression.

Finally, we examined the expression of genes regulated by STAT3 and Nrf2. *Sod2* mRNA expression was upregulated by brusatol (F_BT(2,30)_ = 5.325, *p* = 0.010) and MeHg (F_MeHg(1,30)_ = 9.645, *p* = 0.004) (Fig. 5Q). This increase is concomitant with STAT3 activation, supporting the role of STAT3 as a regulator of *Sod2* gene expression. However, a significant interaction (F_IntM−B(2,30)_ = 7.305, *p* = 0.003) indicated that *Slc7a11* mRNA was inhibited by brusatol in cells exposed to MeHg (Fig. 5R), which parallels the effects of brusatol on Nrf2 protein levels (Fig. 5D and E).

Combined, these data highlight the role of brusatol in modulating key signaling pathways such as STAT3 and Nrf2, offering insights into its potential mechanisms of action. Nrf2 may not be the sole mechanism mediating antioxidant protection, prompting us to test whether STAT3 may serve as an alternative or parallel mechanism that mediates cellular protection against MeHg.

### Nrf2 Pharmacological Activation Induced Antioxidant Gene Expression Without Influencing STAT3 Activation in Response to MeHg Exposure

We demonstrated that pharmacological inhibition of Nrf2 is concomitant to STAT3 activation. To further characterize the interplay between Nrf2 and STAT3, we next assessed whether the opposite scenario is feasible —specifically, whether Nrf2 activation mediates changes in STAT3 phosphorylation. We pharmacologically induced Nrf2 with sulforaphane (SFN), a potent activator of Nrf2, which leads to the induction of phase II detoxification enzymes [59]. The significant increase in LDH release observed when MeHg and 10 µSFN were combined (as indicated by a significant interaction: (F_IntM−S(2,36)_ = 17.475, *p* < 0.001) demonstrated that the co-exposure was more toxic than either compound alone at 6 h (Fig. 6A), suggesting that SFN exacerbated MeHg-induced membrane damage at this early time point. This observation was corroborated by the MTT assay (F_IntM−S(2,36)_ = 14.104, *p* < 0.001) (Fig. 6B), which further supported the conclusion that the combined exposure to MeHg and SFN reduced cell viability to a greater extent than either compound alone, demonstrating a synergistic effect between MeHg and SFN at 6 h. At 24 h, however, the data interpretation became more complex (data not shown). The substantial toxicity of SFN itself at this later time point made it challenging to discern whether the combined effect with MeHg was simply additive or if a synergistic interaction was being masked by SFN’s independent toxicity. As expected, MeHg increased ROS levels at 3 h (F_MeHg(1,30)_ = 30.137, *p* < 0.001) and at 6 h (F_MeHg(1,30)_ = 26.288, *p* < 0.001) (Fig. 6C and D). However, SFN did not affect ROS production. Given the clear evidence of a synergistic interaction between MeHg and SFN at 6 h, this time point was selected for subsequent experiments.

Next, we examined whether SFN modulates MeHg-induced STAT3 activation. Western blot analysis revealed that SFN did not alter the phospho-STAT3/STAT3 ratio (Fig. 7A and B) and no significant changes in SOCS3 protein levels were observed (Fig. 7A, C). SFN increased MeHg-induced Nrf2 protein levels (F_IntM−S(2,30)_ = 6.538, *p* = 0.004) (Fig. 7D and E), whereas no change in KEAP1 protein levels were observed (Fig. 7D, F), supporting that SFN induced Nrf2 by mechanisms independent to KEAP1. To determine if Nrf2 protein induction mediates the activation of antioxidant defense, we measured SOD2 and HO-1 protein levels. Neither MeHg nor SFN affected SOD2 protein levels (Fig. 7G and I). However, MeHg induced HO-1 protein expression (F_MeHg(1,30)_ = 16.062, *p* < 0.001), which was further increased by SFN (F_SFN(2,30)_ = 14.275, *p* < 0.001) (Fig. 7G H), paralleling the changes observed in Nrf2 protein levels (Fig. 7D and E).

As both STAT3 and Nrf2 are transcription factors, we assessed the expression of several of their main target genes using qPCR. In line with the previously described changes in phospho-STAT3/STAT3 ratio *Stat3* expression was increased by MeHg (F_MeHg(1,24)_ = 99.916, *p* < 0.001) (Fig. 7J). Surprisingly, a significant interaction demonstrated that SFN exacerbates MeHg-induced *Socs3* gene expression (F_IntM−S(2,24)_ = 8.031, *p* = 0.002) (Fig. 7K). Interestingly, SFN exacerbated MeHg-induced *Il-6* gene expression, as shown by significant interaction (F_IntM−S(2,24)_ = 4.112, *p* = 0.029) (Fig. 7L). As expected, *Nfe2l2* gene expression was increased by MeHg (F_MeHg(1,24)_ = 24.985, *p* < 0.001) and SFN (F_SFN(2,24)_ = 7.132, *p* = 0.004) (Fig. 7M). While MeHg significantly increased *Keap1* gene expression (F_MeHg(1,24)_ = 6.100, *p* = 0.021), SFN did not exert any significant effect on *Keap1* gene expression (Fig. 7N).

Next, we examined several Nrf2 target genes. While MeHg did not significantly affect *Nqo1*gene expression, the higher dose of SFN increased its expression (F_SFN(2,24)_ = 5.997, *p* = 0.008) (Fig. 7O). In contrast, both MeHg (F_MeHg(1,24)_ = 97.223, *p* < 0.001) and SFN (F_SFN(2,24)_ = 38.403, *p* < 0.001) increased *Hmox1* mRNA levels (Fig. 7P). Neither MeHg nor SFN induced significant changes in *Sod2* gene expression (Fig. 7Q). Notably, a significant interaction demonstrated that SFN enhanced MeHg-induced *Slc7a11 mRNA* expression (F_IntM−S(2,24)_ = 5.124, *p* = 0.014) (Fig. 7R). Together, these data showed that SFN exacerbated MeHg-induced antioxidant gene expression via Nrf2.

To further determine whether Nrf2-responsive signaling could be activated at SFN concentrations that did not produce detectable cytotoxicity, we assessed lower SFN concentrations under the same MeHg co-exposure conditions. Low-dose SFN did not significantly increase LDH release or reduce MTT viability, while increasing Nrf2 and HO-1 protein levels. These findings indicate that SFN-mediated induction of Nrf2-responsive signaling can be dissociated from overt cytotoxicity at lower concentrations. (Supplementary Fig. 1).

## Discussion

ROS are physiologically produced because of metabolic processes and environmental toxicant exposures. To cope with this, cells have developed complex antioxidant defense systems to ensure an adequate response to oxidants ensuring cell survival. In this context, Nrf2 has emerged as an evolutionary conserved intracellular mechanism that protects against various endogenous and chemical-derived stressors, including MeHg [10, 36, 60, 61]. Recently, our group demonstrated that STAT3 may also play a role in controlling MeHg-induced oxidative stress [41]. Thus, the present study represents an initial approach to characterizing the role of Nrf2 and STAT3 signaling pathways and their interplay in the context of MeHg-induced oxidative stress. Our data revealed that STAT3 impairment was associated with increased Nrf2 abundance in the nuclear fraction and enhanced Nrf2 downstream gene levels, whereas the inhibition of Nrf2 caused the induction of STAT3 phosphorylation and transcriptional activity. Combined, these novel results support a model in which Nrf2 and STAT3 exhibit reciprocal and potentially compensatory responses during MeHg-induced injury.

**Fig. 8:**
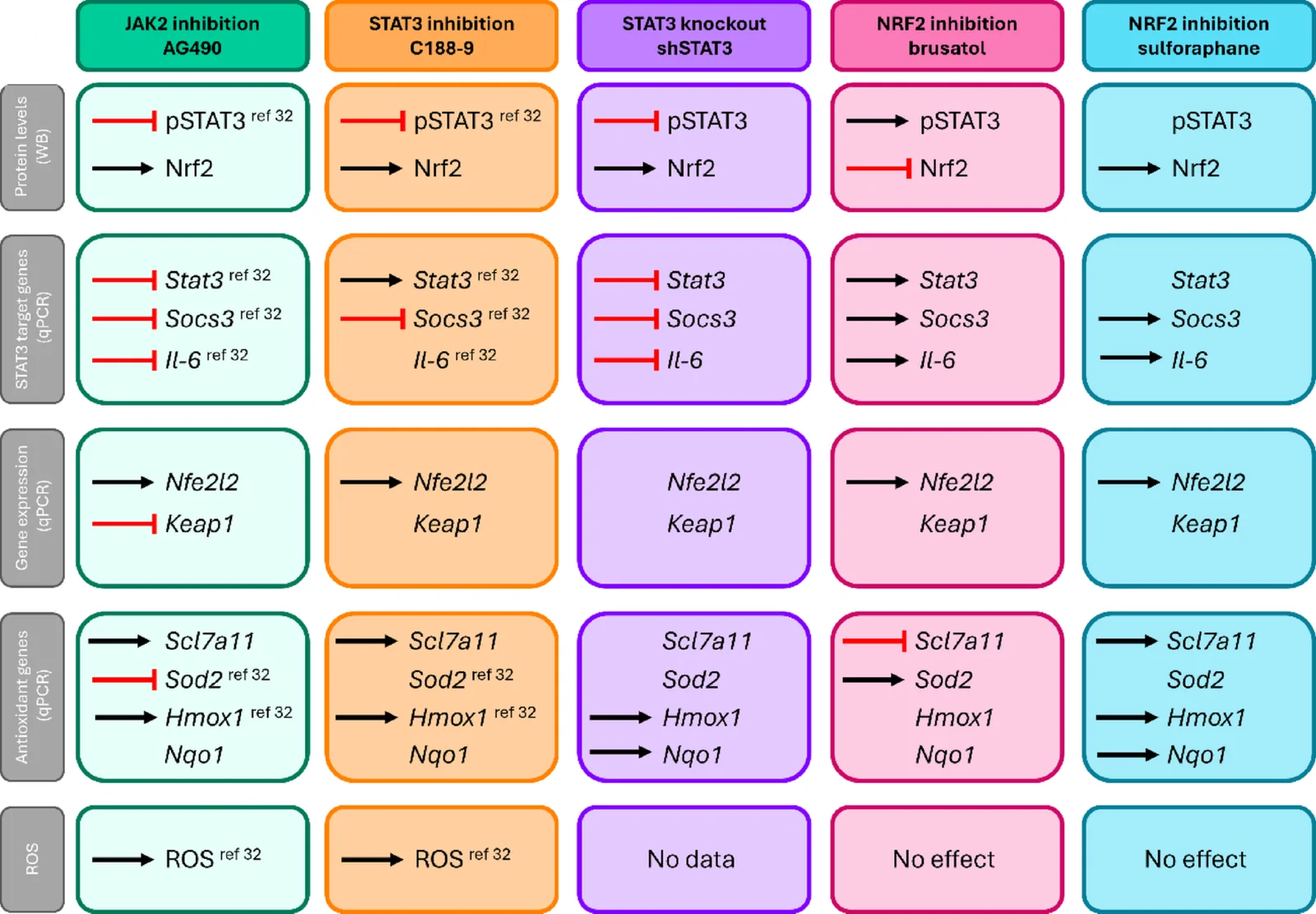
Summary of the effects observed in MeHg-exposed GT1-7 cells across the different experimental conditions. This schematic integrates findings from the present study with previously published results from our group to provide a comprehensive overview of Nrf2- and STAT3-related responses under MeHg exposure. Entries summarize statistically supported changes observed under MeHg exposure alone or under MeHg co-treatment with the indicated pharmacological or genetic intervention. Upward arrows indicate statistically significant increases, downward arrows indicate statistically significant decreases, “—” indicates no statistically significant change, and “ND” indicates not determined. This schematic summarizes experimentally observed changes and does not imply direct molecular interactions

In our previous work [41] we demonstrated that in hypothalamic neuronal GT1-7 cells exposed to MeHg, pharmacological inhibition of STAT3 using AG490 or C188-9 enhanced ROS generation, which was accompanied by an exacerbation of *Hmox1* gene expression, likely as a compensatory mechanism to mitigate the increased oxidative stress. Additionally, treatment with AG490 suppressed *Sod2* mRNA expression, potentially contributing to the increased ROS generation, while C188-9 treatment had no effect on it. Figure 8 provides a comprehensive overview of these findings from our previous work in conjunction with the novel data presented in the current manuscript.

Our data suggest that both Nrf2 and STAT3 signaling pathways may be activated to protect cells against MeHg-induced injury. Notably, concomitant activation of STAT3 and Nrf2 signaling pathways has been reported in other disease models, where oxidative stress plays a crucial role, such as liver fibrosis, lung injury, and hypertensive cardiac pathogenesis [7, 42, 62].

Abrogation of STAT3 phosphorylation exacerbated MeHg-induced Nrf2 activity. Interestingly, several differences emerged depending on the strategy used. For instance, downregulation of STAT3 phosphorylation by inhibiting the upstream kinase JAK2 resulted in the inhibition of *Keap1* gene expression, an effect not observed when STAT3 was genetically ablated nor pharmacologically inhibited at its SH2 domain using C188-9. Although JAK2/STAT3 is the predominant signaling pathway induced by JAK2, it can also bind and activate other members of the STAT family, such as STAT1, STAT2 or STAT5 [63–65]. Therefore, other JAK2-activated pathways may play a role in regulating *Keap1* gene expression. Indeed, Resveratrol protected microglial BV-2 cells against rotenone through inhibition of STAT1 and KEAP1 [66]. Additionally, in aged rats, decreased STAT5b levels were observed concurrently with an increase in KEAP1 protein levels. This increase facilitated Nrf2 cytoplasmic retention, inhibiting Nrf2-mediated antioxidant gene expression, and consequently exacerbating oxidative stress in the liver [67]. As for MeHg’s effects on other members of the STAT family, data have yet to be generated.

When we examined the effects of Nrf2 modulation on STAT3 activity in the context of MeHg exposure, pharmacologically inhibition of Nrf2 using brusatol significantly increased STAT3 activity, whereas pharmacologically activation of Nrf2 with SFN did not affect STAT3 phosphorylation. Previous studies using different carcinoma cells models have reported that brusatol suppressed STAT3 activity [68–70]. However, in our study, Nrf2 inhibition by brusatol in hypothalamic GT1-7 neuronal cells exposed to MeHg resulted in an increased pSTAT3/STAT3 ratio and enhanced STAT3 transcriptional activity. These discrepancies suggest that the effects of brusatol may be cell type-dependent with its impact on STAT3 activity varying based on the cellular context and model.

Nonetheless, brusatol did not alter ROS production in MeHg-exposed cells, other than the decrease in Nrf2 protein levels. These findings are consistent with several other studies that have demonstrated a reduction in Nrf2 protein levels upon brusatol treatment without concomitant alterations in cellular ROS levels [69, 71]. Here, the inability of brusatol to exacerbate MeHg-induced ROS production, despite Nrf2 inhibition, may be explained by the lack of inhibition on several Nrf2-induced antioxidant genes and the concomitant activation of STAT3 transcriptional activity and upregulation of *Sod2* gene expression, a gene transcriptionally regulated by STAT3. Taken together, these findings suggest that additional Nrf2-independent mechanisms may partially compensate for impaired Nrf2 signaling during MeHg-induced oxidative stress. In this context, our data support STAT3 activation as a potential alternative protective mechanism. Additionally, under basal conditions, the brusatol-induced increase in STAT3 activity was accompanied by a decrease in SOCS3 protein levels, suggesting that one of the mechanisms which mediated brusatol’s effects on STAT3 activation is the repression of STAT3 inhibitors, such as SOCS3.

Surprisingly, while brusatol decreased Nrf2 protein levels in cells exposed to MeHg, the *Nfe2l2* mRNA expression was increased, possibly acting as a compensatory mechanism to counteract Nrf2 protein decrease. The brusatol activation of *Nfe2l2* mRNA has been previously reported [72, 73]. Our data further supports this finding, showing that brusatol increased *Nfe2l2* mRNA expression. The absence of change in KEAP1 and the observation that the decrease in Nrf2 protein levels was not accompanied by a decrease in *Nfe2l2* mRNA expression suggests that brusatol may repress the translation of Nrf2. Indeed, a mechanism of Nrf2 translational repression, involving an open reading frame of this gene, has been identified [74]. Further research is needed to clarify this hypothesis.

As it was previously demonstrated in vivo and in vitro studies, SFN mediated activation of Nrf2 pathway, leading to the induction of antioxidant proteins. SFN upregulates Nrf2 protein levels by inducing conformational changes in KEAP1 [75] and inhibiting Nrf2 ubiquitination and degradation [76, 77]. MeHg-induced Nrf2 protein accumulation was most clearly demonstrated in the time-course experiment shown in Fig. 1. In the SFN co-treatment experiment shown in Fig. 7, densitometric quantification supported the reported effect on Nrf2 protein levels. In our study, SFN was used as a pharmacological activator of Nrf2-responsive signaling to assess whether Nrf2 activation modulates STAT3 phosphorylation and antioxidant responses under MeHg exposure. Although SFN increased Nrf2 protein expression and upregulated downstream antioxidant genes in GT1-7 cells, it did not reduce MeHg-i This contrasts with previous studies reporting that SFN-mediated Nrf2 activation decreases oxidative stress in other experimental models [78–81], suggesting that the antioxidant effects of SFN may be context-, cell type-, and exposure condition-dependent. Moreover, SFN-induced Nrf2 failed to alter the MeHg-induced STAT3 activation. Similarly, in pancreatic cancer cells, SFN was unable to alter STAT3 phosphorylation [82]. However, these findings contradict several studies where SFN inhibition of the STAT3 signaling pathway has been reported, both in vitro and in vivo models [80, 81, 83–86]. The reason for these discrepancies is not clear. Nevertheless, in some of those studies, SFN-mediated inhibition of STAT3 phosphorylation was proposed to involve SFN-induced ROS accumulation. Therefore, in our study, the lack of SFN effects on STAT3 phosphorylation is likely related to the absence of changes in ROS accumulation.

Although SFN did not affect MeHg-induced STAT3 activation, SFN enhanced the gene expression of *Socs3*. These changes paralleled the alterations observed in Nrf2 protein and gene expression. In hepatic stellar cells, Nrf2 overexpression was shown to increase SOCS3 levels [87]. Additionally, FGF21 treatment diminished liver fibrosis by elevating both Nrf2 and SOCS3 [88]. Similar results were observed in a gonad injury study, where treatment with methylseleninic acid alleviated testicular damage by inducing Nrf2 and SOCS3 [89]. Combined, these observations support the existence of an interplay between Nrf2 and STAT3 signaling pathways.

Our findings highlight a complex regulatory relationship between the STAT3 and Nrf2 pathways in modulating oxidative stress. *Slc7a11* encodes the xCT protein, cysteine/glutamate antiporter that plays a critical role in maintaining redox homeostasis. Interestingly, inhibition of STAT3 increased *Slc7a11* expression, suggesting that STAT3 may act as a negative regulator of this gene under MeHg exposure. However, in STAT3KO cells exposed to MeHg, while *Hmox1*mRNA expression is exacerbated, *Slc7a11* mRNA levels remained similar to those observed in MeHg-exposed control cells. These discrepancies suggest that STAT3 may partially regulate *Slc7a11*. In fact, previous studies show that STAT3 can regulate xCT [90]. Furthermore, inhibition of Nrf2 using brusatol was associated with increased STAT3 activation. However, this increase in STAT3 was not sufficient to restore *Slc7a11* mRNA expression following Nrf2 inhibition in MeHg exposed cells. Thus, STAT3 may play a role in further activating *Slc7a11* expression rather than in initiating its activation. These findings suggest that, in the context of MeHg-induced neurotoxicity, STAT3 may play a modulatory role in regulating oxidative stress responses, acting to reinforce rather than replace Nrf2.

In addition to oxidative stress, inflammation has been implicated in the pathogenesis of MeHg-induced neurotoxicity. Epidemiologic studies have established a correlation between MeHg exposure and immune system dysfunction [91]. Moreover, in vitro and in vivo studies have demonstrated that MeHg alters the cytokine expression profile. Among these, IL-6 is the most studied cytokine. MeHg has been shown to increase IL-6 protein and mRNA levels in various glial cells cultures from different species [92–94] and in different in vivo models [95–97]. Nevertheless, other studies reported a decline in this cytokine levels in Swiss mice exposed to high doses of MeHg [98] and in primary microglial cultures from BALB/C neonatal brains [99]. The reasons for these discrepancies are unclear, but one explanation may be the different exposure paradigms and experimental models. Our data demonstrate that MeHg induced an increase in *Il-6* gene expression, consistent with observations in SH-SY5Y neuronal cell cultures [95]. Notably, inhibition of MeHg-induced Nrf2 activation with brusatol induced *Il-6* mRNA expression, concomitant with an increase in pSTAT3/STAT3 ratio. In contrast, SFN increased *Il-6* gene expression without affecting STAT3 activation. Interestingly, the JAK2 inhibitor AG490 ameliorated MeHg-induced *Il-6* mRNA expression, while STAT3 inhibitor C188-9 had no effect on its expression [41]. Although IL-6 is predominantly regulated by the STAT3 pathway, our findings raise the hypothesis that alternative JAK2-mediated mechanisms may contribute to MeHg-induced *IL-6* gene expression. In glial cells, phospholipase A2 and C are required for MeHg-induced IL-6 release [100, 101]. In studies of inflammatory process in alveolar epithelial cells, the inhibition of JAK2 with AG490 led to decreased phospholipase A2 [102] and C [103] activity. Although not directly tested in the present study, these observations raise the possibility that MeHg-induced IL-6 expression may involve phospholipase A2 and/or C signaling, potentially through mechanisms not limited to canonical STAT3 activation. Future studies will be required to directly test this hypothesis.

Inhibition of Nrf2 has been shown to be accompanied by increased STAT3 phosphorylation levels, and the vice versa [42, 44, 45, 62, 86, 104]. Furthermore, the existence of AREs, the Nrf2 binding sequence, in pro-inflammatory genes such as *Il-6* has been reported [105]. Similarly, STAT binding elements (SBE) sequence, exists in antioxidant genes like *Sod2* [17]. Considering these data, a potential interplay between these two transcription factors may exist. Computational studies based on domain-motif interactions have predicted a possible connection between Nrf2 and STAT3 [106]. Indeed, in breast cancer cells STAT3 has been shown to promote Nrf2 nuclear localization via direct interaction. STAT3 may protect Nrf2 from KEAP1-mediated degradation, stabilizing Nrf2 in the nucleus [107].

It is noteworthy that MeHg can also activate nuclear factor kappa B (NF-κB), which in turn induces the expression of chemokines [108, 109] and cytokines, including IL-6 [110]. In fact, the p50 and p65 NF-κB subunits physical interact with STAT3 [111]. Interestingly, a crosstalk between Nrf2 and NF-κB has been also reported [112–114]. By binding to the CH1-KIX domain of CREB binding protein (CBP), NF-κB p65 subunit disrupts the interaction between CBP and Nrf2, thereby inhibiting Nrf2’s transcriptional activity [115]. Although NF-κB was not directly assessed in the present study, based on these published observations, it is reasonable to hypothesize that NF-κB, STAT3, and Nrf2 form an interconnected regulatory network, which coordinates antioxidant and inflammatory responses. Therefore, we cannot rule out that some of the observed results are influenced by the NF-κB signaling pathway. Future studies directly assessing NF-κB activation will be needed to clarify this possibility.

Despite the strengths of our study, several limitations should be acknowledged. First, the use of an in vitro model base on GT1-7 hypothalamic neuronal cells, while valuable for dissecting specific molecular responses, cannot fully replicate the complexity of in vivo neuronal systems. Studies in additional cell lines and/or in vivo studies are necessary to further validate our findings. Second, although our results demonstrate that both STAT3 and Nrf2 signaling pathways are activated in response to MeHg and function as a protective mechanism, the full molecular basis of their interaction remains to be defined. Notably, we observed that inhibition of one pathway leads to activation of the other, suggesting a potential regulatory crosstalk aimed at maintaining redox homeostasis under MeHg exposure. However, whether this interplay occurs similarly in vivo or across different cell types has yet to be determined. Additionally, while we used genetic approaches to manipulate STAT3, Nrf2 was only modulated pharmacologically. Moreover, although brusatol is widely used as an Nrf2 inhibitor, its effects may extend beyond selective Nrf2 inhibition and may include Nrf2-independent or off-target actions. Future studies using genetic approaches to manipulate Nrf2 expression will help to further elucidate the interplay between STAT3 and Nrf2. Overall, our findings should be viewed as an initial framework for future investigations. In vivo studies, complementary cell models, and more detailed mechanistic analysis will be essential to validate this compensatory response and to elucidate the coordinated roles of STAT3 and Nrf2 in neuronal defense against MeHg-induced neurotoxicity.

## Conclusion

In conclusion, our findings highlight a reciprocal and potentially compensatory relationship between Nrf2 and STAT3 in response to MeHg exposure. Inactivation of STAT3 or Nrf2 exacerbated MeHg-induced injury and was associated with increased activation of the other pathway, likely as a compensatory mechanism. Furthermore, although lower concentrations of SFN induced Nrf2-responsive signaling without detectable cytotoxicity, this was not accompanied by a clear reduction in MeHg-induced ROS production or cytotoxicity. These findings suggest that additional mechanisms may contribute to the cellular response to MeHg-induced oxidative stress. A better understanding of the interplay between these pathways could aid in the development of new therapeutic strategies for combating MeHg-induced injury.

## Supplementary Information

Below is the link to the electronic supplementary material.

Supplementary Material 1. Supplementary Fig: 1. Sulforaphane (SFN) induces Nrf2 and HO1 within a better-tolerated dose range in methylmemercury (MeHg)- exposed mouse hypothalamic neuronal GT1-7 cells. **A** Cytotoxicity measured by LDH release in GT1-7 cells pre-treated 30 min with 1, 2, 5, or 10 µM SFN, followed by co-exposure to 5 µM MeHg for 6 h (*n* = 6 independent cell culture preparations). **B** Cell viability assessed by MTT assay in GT1-7 cells pre-treated 30 min with 1, 2, 5, or 10 µM SFN, followed by co-exposure to 5 µM MeHg for 6 h (*n* = 6 independent cell culture preparations). For western blot studies, GT1-7 cells were pre-treated 30 min with 2, 5, or 10 µM SFN followed by 6 h co-exposure to 5 µM MeHg. **C** Representative western blot images of phospho-STAT3 (Y705), total STAT3, Nrf2, and β-actin. **D** Densitometry quantification of phospho-STAT3/STAT3 ratio (*n* = 6 independent cell culture preparations). **E** Densitometry quantification of Nrf2 protein levels (*n* = 6 independent cell culture preparations). **F** Representative western blot images of HO-1 and β-actin. **G** Densitometry quantification of HO-1 protein levels (*n* = 6 independent cell culture preparations). Data are expressed as mean ± SD. * indicates *p* < 0.05

## Data Availability

The data that support the findings of this study are available from the corresponding author upon reasonable request.

## Abbreviations

HO-1: Heme oxygenase 1
*Hmox1*: Heme oxygenase 1 gene
IL-6: Interleukin 6
JAK2: Janus kinase 2
KEAP1: Kelch-like ECH-associated protein 1
MeHg: Methylmercury
NQO1: NAD(P)H dehydrogenase quinone 1
Nrf2: Nuclear factor erythroid 2-related factor 2
*Nfe2l2*: Nuclear factor erythroid 2-related factor 2 gene
ROS: Reactive oxygen species
SLC7a11: Solute carrier family 7 member 11
SFN: Sulforaphane
SOCS3: Suppressor of cytokine signaling 3
SOD2: Superoxide dismutase 2
STAT3: Signal transducer and activator of transcription 3
