# Supplementary material for: Nrf2 and STAT3 Pathways in Protecting Against Methylmercury-Induced Oxidative Stress in Hypothalamic Neuronal GT1-7 Cells
Source: Neurochem Res. 2026 Oct 8;51(5):283. doi: 10.1007/s11064-026-04898-1 (PMC13649924; doi:10.1007/s11064-026-04898-1)
Supplement: Supplementary file 1 [file 11064_2026_4898_MOESM1_ESM.pdf]

Supplementary Figure 1

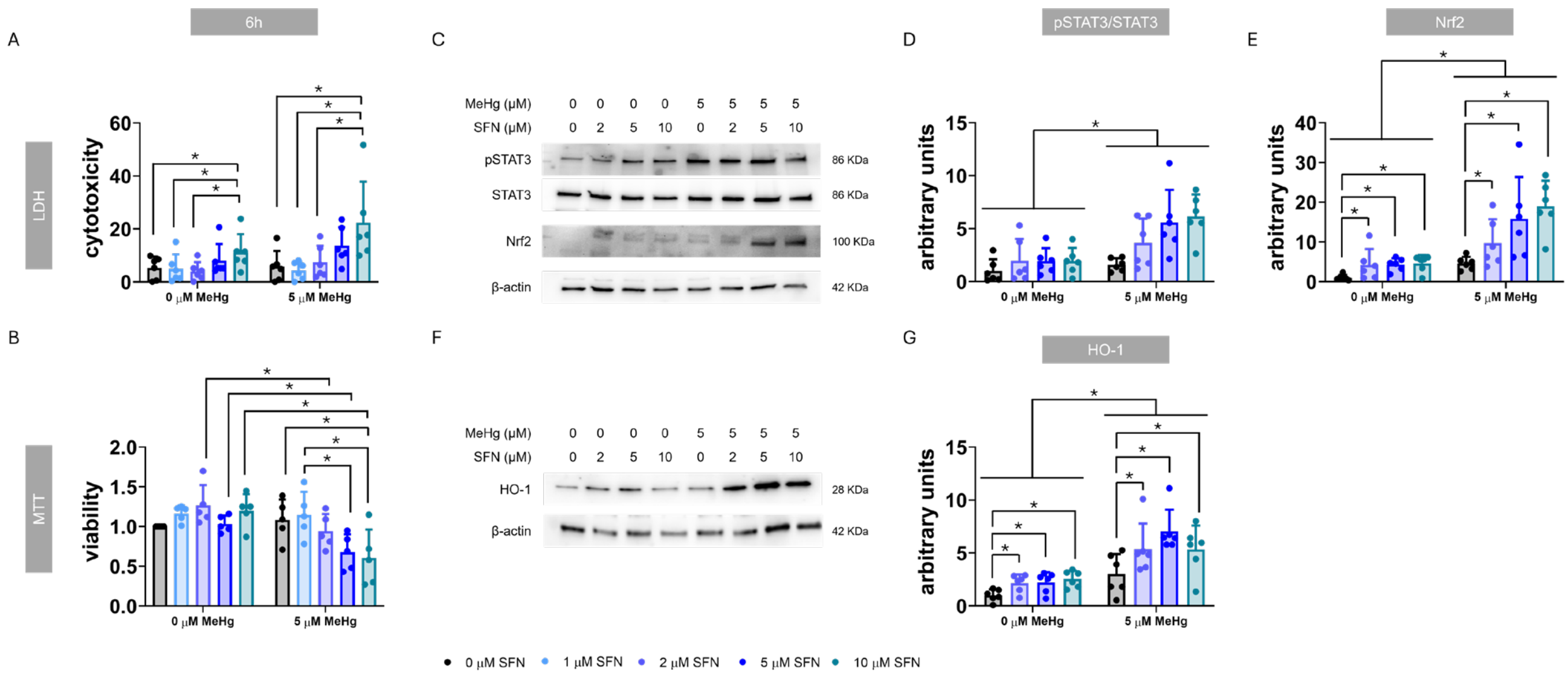

Supplementary Fig. 1. Sulforaphane (SFN) induces Nrf2 and HO1 within a better-tolerated dose range in methylmemercury (MeHg)- exposed mouse hypothalamic neuronal GT1-7 cells. A) Cytotoxicity measured by LDH release in GT1-7 cells pre-treated 30 min with 1, 2, 5, or 10 µM SFN, followed by co-exposure to 5 µM MeHg for 6 hours (n=6 independent cell culture preparations). B) Cell viability assessed by MTT assay in GT1-7 cells pre-treated 30 min with 1, 2, 5, or 10 µM SFN, followed by co-exposure to 5 µM MeHg for 6 hours (n=6 independent cell culture preparations). For western blot studies, GT1-7 cells were pre-treated 30 min with 2, 5, or 10 µM SFN followed by 6 hours co-exposure to 5 µM MeHg. C) Representative western blot images of phospho-STAT3 (Y705), total STAT3, Nrf2, and β-actin. D) Densitometry quantification of phospho-STAT3/STAT3 ratio (n=6 independent cell culture preparations). E) Densitometry quantification of Nrf2 protein levels (n=6 independent cell culture preparations). F) Representative western blot images of HO-1 and β-actin. G) Densitometry quantification of HO-1 protein levels (n=6 independent cell culture preparations). Data are expressed as mean ± SD. * indicates $p < 0.05$.
